# All Nanozyme‐Based Cascade Reactions for Biomedical Applications: from Self‐Cascading Nanozyme to Immobilized Cascade Nanozyme

**DOI:** 10.1002/advs.202519656

**Published:** 2025-12-12

**Authors:** Caixia Zhu, Congcong Jiang, Hian Kee Lee, Yuanjian Zhang, Sheng Tang

**Affiliations:** ^1^ School of Environmental and Chemical Engineering Jiangsu University of Science and Technology Zhenjiang 212003 China; ^2^ Department of Chemistry School of Chemistry and Chemical Engineering National University of Singapore 3 Science Drive 3 Singapore 117543 Singapore; ^3^ Jiangsu Engineering Research Center of Smart Carbon‐Rich Materials and Devices School of Chemistry and Chemical Engineering Nanjing 211189 China; ^4^ Department of Oncology, Zhongda Hospital Southeast University Nanjing 210009 China

**Keywords:** biomedical applications, cascade reaction, catalytic mechanisms, nanozyme

## Abstract

As artificial substitutes for natural enzymes, nanozymes possess advantages such as high catalytic activity, low cost, excellent stability, and suitability for large‐scale production. Inspired by the cascade reactions in biological systems, constructing cascade nanozyme systems with step‐saving and high efficiency has been recognized as a key approach to enhancing the functional performance of nanozymes. With the discovery of more nanomaterials with various enzyme‐like activities, especially the unique multi‐enzyme activity of nanozymes, unprecedented opportunities have arisen for advancing biomimetic design to a higher level. Furthermore, aided by advanced tools such as theoretical calculations, the structural design and functional tuning of nanozymes have gradually become customizable and intelligent, significantly promoting their application in specific tasks ranging from biosensing to therapy. This review introduces the evolution of all‐nanozyme cascade reaction systems from a self‐cascading nanozyme system to immobilized nanozyme‐based cascade catalytic system, and introduces key mechanistic insights and commonly used research methods to clarify their catalytic characteristics and design principles. A detailed classification of all‐nanozyme cascade reaction systems is provided, and an analytical survey of recent applications of all‐nanozyme cascade reaction systems in biosensing and therapy is covered. Finally, this review discusses the challenges that all‐nanozyme cascade reaction systems may face in their application.

## Graphical abstract

This review introduces the development and classification of all‐nanozyme cascade reaction systems, highlighting mechanistic insights and research methods for understanding catalytic features and design principles. It provides detailed classifications, surveys recent applications in biosensing and therapy, and discusses the challenges that all‐nanozyme cascade reaction systems may face in their application.

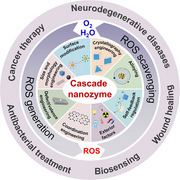

## 1 Introduction

During the early stages of life, natural enzymes supplanted primitive inorganic catalysts because they provided markedly higher catalytic rates, greater substrate specificity, and the capacity to catalyze reactions under mild physicochemical conditions (ambient temperature, atmospheric pressure, aqueous medium).^[^ ^1^ ^]^ Consequently, enzymes became the principal catalytic components of metabolic networks by virtue of natural selection. However, a single enzyme typically catalyzes only one chemical transformation step within a metabolic pathway, making it difficult to independently accomplish complex synthetic or metabolic processes requiring sequential multi‐step reactions. For instance, glycolysis, as a paradigmatic metabolic pathway, necessitates the sequential cooperation of multiple enzymes to gradually convert glucose into pyruvate while releasing energy;^[^ ^2^ ^]^ this demonstrates the necessity of multienzyme coordination for many fundamental metabolic processes. Moreover, at the level of signal transduction, multistep enzymatic cascades such as mitogen‐activated protein kinase (MAPK) pathway through a series of enzymes that activate or inhibit each other to amplify and regulate signals.^[^ ^3^ ^]^ These integrative regulatory functions are not attainable by single catalytic elements. Therefore, the limitations of single‐enzyme catalysis in terms of functional scope and robustness have driven biological systems to universally adopt cascade catalysis as the primary mode for achieving complex metabolic and signaling functions.

Cascade reaction refers to a catalytic process integrating at least two consecutive reactions in which each subsequent reaction is driven by the transformation of the preceding one, requiring the synergistic effect of multiple enzymes. In living organisms, enzyme cascade systems have been evolved by two complementary strategies, i.e., compartmentalization and substrate channeling for constructing multi‐enzyme catalytic systems with superior catalytic activity and stabilities in practical biocatalysis. Owing to their step‐saving, high efficiency, and avoidance of separation of the intermediate, enzyme cascade reactions have attracted special attention from both biologists and chemists. Initially, researchers tended to use free enzymes to form simple enzyme cascade catalytic systems,^[^ ^4^, ^5^, ^6^, ^7^ ^]^ eliminating the tedious isolation and purification of reaction intermediates, thereby directly reducing costs and waste of the transformations. To further mimic enzyme cascade systems of living organisms, extensive research efforts have been directed toward reducing mass transfer distance and improving catalytic efficiency by developing biocatalytic cascades in confined micro or nano environments,^[^ ^8^, ^9^ ^]^ or by loading biocatalysts onto scaffolds.^[^ ^10^, ^11^, ^12^ ^]^ In recent years, owing to their high efficiency and signal amplification capabilities, enzyme cascade systems have been extensively utilized in the field of therapy and biosensing.^[^ ^13^ ^]^ However, the chemical structure of biological macromolecules results in inherent drawbacks such as low stability, high cost, and low tolerance to harsh environments, which have restricted the performance of enzymes in biomedical applications. To overcome these shortcomings, the development of artificial enzymes is critical for creating innovative cascade catalytic systems.

As next‐generation artificial enzymes, nanozymes are nanomaterials with intrinsic enzyme‐like characteristics. Since the discovery of the peroxidase (POD)‐like activity of Fe_3_O_4_ nanoparticles in 2007,^[^ ^14^ ^]^ nanozymes have become an emerging alternative to natural enzyme for numerous important applications due to their superior stability and cost‐effective. Importantly, unlike natural enzymes that typically possess a single catalytic function, many nanozymes demonstrate multi‐enzyme activity at a single active site, which provides the opportunity for the development of biomimetic self‐cascade reaction systems. The application of multienzyme‐like nanozymes in cascade sensing systems not only enhances reaction kinetics but also promotes signal transduction and amplification.^[^ ^15^, ^16^, ^17^, ^18^, ^19^ ^]^ In recent years, aided by advanced tools such as theoretical calculations, the structural design and functional tuning of nanozymes have gradually become customizable and intelligent. To improve the efficiency of cascade reactions, researchers have increasingly focused on synthesizing functionally tunable immobilized nanozymes to construct nanozyme cascade systems. Immobilized nanozymes obtain enhanced cascade reaction activity through the synergistic action of multiple active sites by integrating multiple different enzyme‐like components. It has been demonstrated that the spatial limitation of immobilization significantly minimizes intermediate diffusion of nanozymatic cascade reaction and improves overall catalytic efficiency.^[^ ^20^, ^21^, ^22^ ^]^ As a result, nanozyme‐based cascade systems offer several distinct advantages, including enhanced reusability, increased selectivity, and more orderly signal amplification. These distinctive features enable the nanozyme system to demonstrate broad application prospects and development potential in multi‐step cascade catalysis, high‐sensitivity biosensing, and other advanced biomimetic catalytic applications.^[^ ^23^, ^24^, ^25^, ^26^ ^]^

The application of nanozyme‐based cascade reaction system has attracted extensive attention from researchers, and several reviews on nanozyme‐involved cascade reaction system have been published. Recently, some of these reviewed nanozyme‐involved cascading reaction system in biosensing,^[^ ^27^ ^]^ food quality detection,^[^ ^28^ ^]^ cancer therapy,^[^ ^29^ ^]^ and environmental analysis.^[^ ^30^ ^]^ However, cascade reaction systems that couple natural enzymes constitute a significant proportion of these research articles. Since nanozymes have positioned themselves as important candidates for natural enzymes, a review focusing on the construction of all‐nanozyme cascade reaction, without enzyme, will help in advancing the field. In addition, most of the previous reviews did not provide a comprehensive overview of all‐nanozyme‐based cascade forms. Herein, this review categorically summarizes the all‐nanozyme cascade system and presents their applications in biomedicine. First, the classification standard of cascade nanozyme is introduced, followed by the classification and elaboration on the all‐nanozyme cascade system. We then focus on commonly used strategies for improving catalytic efficiency and their design principles. Thereafter, a detailed classification of all‐nanozyme cascade reaction systems is provided, and a summary of recent applications of all‐nanozyme cascade reaction systems in biosensing and therapy is presented. Finally, current challenges and future opportunities of constructing all‐nanozyme cascade reaction systems with high catalytic efficiency are presented.

## 2 Construction of Cascade Catalytic Systems

Enzyme cascades have received considerable attention in the biomedical field, benefiting from their advantage of high efficiency, step‐saving, and signal amplification. In recent years, many cascade catalytic systems composed of natural enzymes and nanozymes have been reported.^[^ ^31^, ^32^, ^33^ ^]^ However, compared with the system involving natural enzymes, the all‐nanozyme cascade system exhibits obvious advantages, such as low cost, controllable activity and good stability.^[^ ^34^, ^35^ ^]^ Moreover, most nanozymes have unique characteristics of multiple enzyme activities, providing new opportunities for constructing high‐efficiency cascade reaction systems.

Prior to this review proper, it is essential here to clarify the definitions of self‐cascading and immobilized nanozymes. According to the source of catalytic activity of nanozymes, cascade nanozymes can be categorized into self‐cascading and immobilized types. The self‐cascading nanozyme refers to a single nanostructure with multiple enzymatic catalytic functions at the same catalytic site. A defining characteristic is a single active site that can mimic the activity of different enzymes. Immobilized cascade nanozymes integrate multiple different enzyme‐like components and have multiple active sites. Another major feature of this type of cascade nanozyme is its rational designability, along with the enhanced cascade reaction activity achieved through the synergistic effects of multiple confined catalytic sites.

### 2.1 Self‐Cascading Nanozyme‐Based Cascade Catalytic System

**Figure 1:**
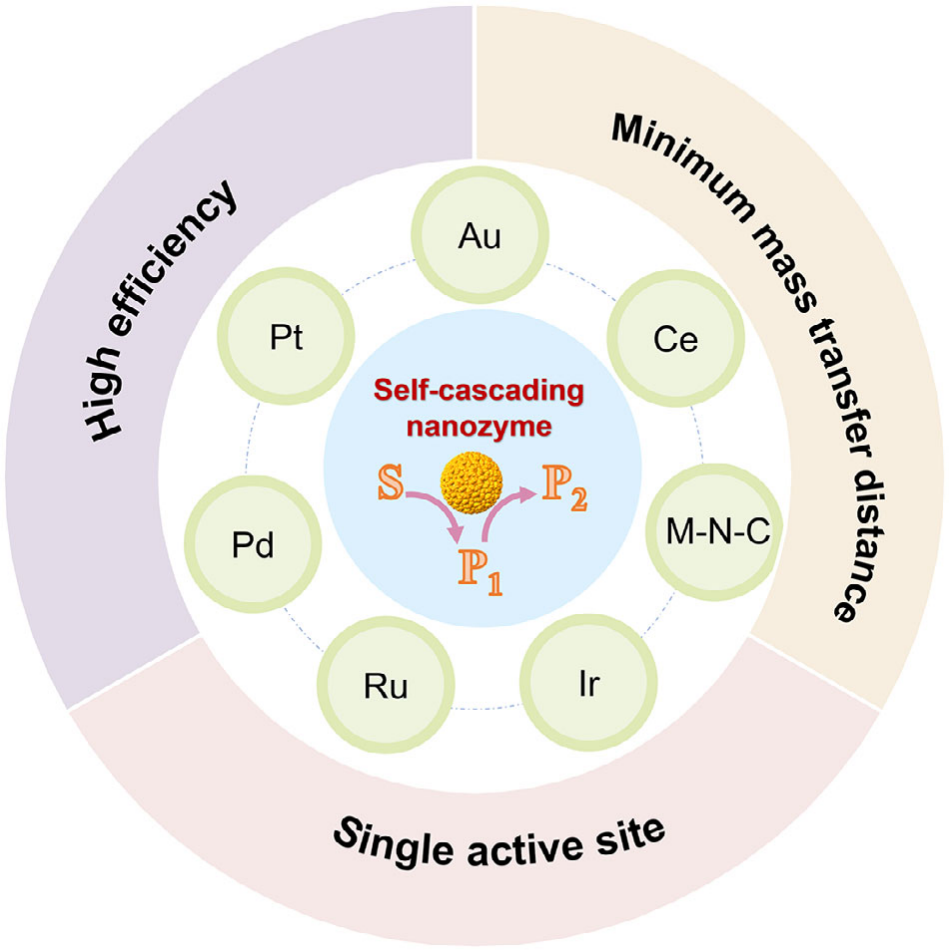
Schematic of characteristics and composition of self‐cascading nanozymes.

Self‐cascading nanozymes mainly exhibit multiple enzymatic activities in one active center,^[^ ^36^ ^]^ minimizing the mass transfer distance in cascade reaction (**Figure** **1**). Utilizing self‐cascade nanozymes as the central components, thereby simplifying system composition, has become an important strategy in the construction of all‐nanozyme‐based cascade systems in recent years.^[^ ^37^, ^38^, ^39^, ^40^ ^]^ As a typical example, cerium‐based nanomaterials, owing to their abundant Ce^3+^/Ce^4+^ redox couple, exhibit multiple enzyme‐mimicking activities. For example, Jin et al. proposed a mild photothermal‐enhanced cascaded nanozyme‐catalyzed therapy strategy based on the cascade nanozyme (Ce SAs@NC) with POD, catalase (CAT), and oxidase (OXD) activities.^[^ ^41^ ^]^ Ce SAs@NC leverages its high Ce^3+^/Ce^4+^ ratio and atomic‐level active sites to catalyze hydrogen peroxide (H_2_O_2_) decomposition into cytotoxic hydroxyl radicals (•OH) and O_2_, followed by the conversion of O_2_ into superoxide anion free radical (O_2_^•−^), while its photothermal properties further amplify enzyme activity under near‐infrared (NIR) light, enabling efficient tumor inhibition through combined nanozyme‐catalyzed therapy (NCT) and immune modulation.

Au NPs are usually selected as one primary component for catalyst design for the glucose cascade reaction due to its intrinsic GOD‐like and POD‐like activity. For example, Qian et al prepared tannic acid‐modified gold nanoflowers (TA@AuNFs) by using TA as a reducing agent, which presented POD‐like and glucose oxidase (GOx)‐like activities owing to the abundant galloyl residues on the surface of AuNFs.^[^ ^42^ ^]^ Similarly, Dai et al. constructed a metalloprotein‐like Au nanozyme (ANZ) capable of self‐cascade ROS generation without external H_2_O_2_, enabling potent antibacterial activity against drug‐resistant pathogens.^[^ ^43^ ^]^ Overall, these Au‐based self‐cascading nanozymes show diverse functions and broad application prospects in disease treatment and biological detection.

It has also demonstrated that subordinative platinum group metal‐based (e.g., Pt, Ir, Ru, and Pd) nanomaterials exhibit multifunctional enzyme‐mimetic behaviors, notably POD‐like, CAT‐like and superoxide dismutase (SOD)‐like activities.^[^ ^44^, ^45^, ^46^, ^47^ ^]^ For example, Wang et al. synthesized ultrasmall ruthenium nanoparticles (RuNPs) with multi‐type of enzymatic activities to eliminate both ROS and reactive nitrogen species (RNS) for preventing postoperative peritoneal adhesion.^[^ ^48^ ^]^ The activities of these types nanomaterials can probably be ascribed to readily accessible redox couples at the metal surface and to facile adsorption/desorption of oxygenated intermediates. In another example, Zheng et al. reported a laminarin‐modified platinum nanozyme (Pt@LA) to serve as stable and active nanozymes with self‐cascade SOD‐like and CAT‐like catalytic activities.^[^ ^49^ ^]^ These Pt nanozymes featured ultra‐small size (3–4 nm) that promotes surface electron transfer and catalytic synergy.

In addition, some metal‐nitrogen‐carbon (M‐N‐C) materials possess multiple enzyme‐like activities depending on surface functional groups, defect density, and heteroatom doping. For instance, Zhang et al. developed Cu‐based single‐atom nanozymes (Cu‐NS SA) with an asymmetric coordination environment (Cu‐N_3_S_1_).^[^ ^50^ ^]^ Compared with symmetric Cu‐N_4_ single‐atom nanozymes, the Cu‐NS SA nanozyme exhibited more excellent enzyme‐like properties, including POD, NADPH oxidase (NOX), L‐cysteine oxidase (LCO), and glutathione oxidase (GSHOx), which not only cause enough reactive oxygen species (ROS) storms through cascade reaction, but also consume antioxidative cellular metabolisms to trigger pyroptosis. Recently, Huang et al. developed a strategy to synthesize axially chlorinated single‐atom Fe/N‐doped carbon nanodots (Cl‐FeNCDs) via a one‐pot solvothermal method using hemin as the precursor.^[^ ^51^ ^]^ Cl‐FeNCDs exhibit integrated SOD‐like, POD‐like, and myeloperoxidase (MPO)‐like activities, enabling a light‐triggered, neutrophil‐mimetic enzymatic cascade even under hypoxic conditions. By promoting endogenous H_2_O_2_ generation and directing its selective conversion into cytotoxic HClO, the nanozyme achieved self‐cascade amplification for tumor pyroptosis and immunotherapy, particularly when embedded in injectable hydrogels for localized post‐surgical treatment.

### 2.2 Immobilized Nanozyme‐Based Cascade Catalytic System

**Figure 2:**
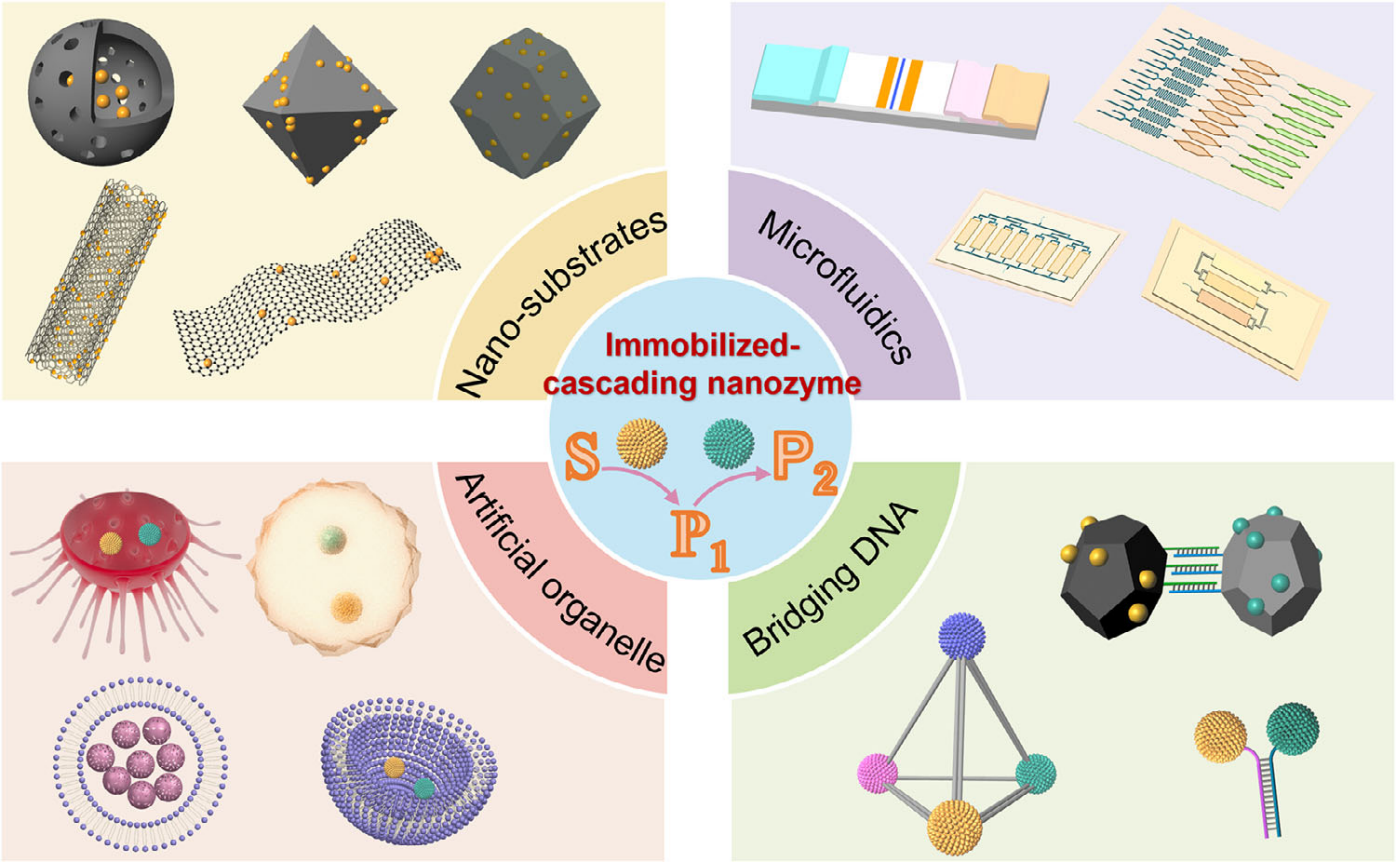
Overview of assembly modes of immobilized nanozyme cascade reaction systems.

The overall catalytic efficiency in cascade reactions is highly influenced by the mass transfer distance of the intermediates. Therefore, strategies that streamline reaction steps and minimize free diffusion are essential for enhancing the cascade reaction property. Inspired by the high efficiency of confined cascade reactions in biological systems, researchers have increasingly focused on designing confined reaction spaces to enhance the efficiency of nanozyme‐based cascade systems. By immobilizing nanozymes in these confined spaces, the distance between different enzyme‐mimicking sites is significantly reduced, increasing the probability that intermediate products from one enzymatic reaction are immediately utilized as substrates by the next enzyme, thereby minimizing intermediate loss. The precise spatial control accelerates reaction rates, reduces undesired side reactions, and limits the accumulation of inhibitory or reactive intermediates. In this section, we provide an overview of various forms of immobilized nanozyme cascade reaction systems, highlighting their potential advantages in optimizing catalytic performance and stability (**Figure** **2**).

#### 2.2.1 Nanozymes Immobilized in Nano‐Substrate

To obtain a stable cascade nanozymes system, an effective method is to immobilize different nanozyme components in one nano‐substrate. Metal‐organic frameworks (MOFs) are ideal carriers for self‐assembling nanozymes because of their large surface areas, well‐defined pore structures, tunable chemical composition, and multi‐functional sites. As a typical example, He et al. developed a hybridized nanozyme (AuNPs@PCN‐224(Fe)) by growing ultrasmall AuNPs in the internal cavities of an iron (Fe) porphyrin‐based MOF.^[^ ^52^ ^]^ Based on the bifunctional AuNPs@PCN‐224(Fe), in which AuNPs and PCN‐224(Fe) exhibited the catalytic activity of GOx and POD, respectively, a self‐cascade catalytic colorimetric detection system was constructed for glucose detection. Furthermore, the distinctive capability of a MOF structure to link metal nodes possessing diverse catalytic activities via ligands allows for the design and fabrication of MOFs with multienzyme‐like activities. For example, Liu et al. prepared a dual‐Fe‐atom nanozyme (Fe_2_NC) by using abundant anchor sites on the ZIF‐8‐derived nitrogen‐doped carbon material to stabilize binuclear Fe_2_ sites. Following the successful synthesis of Fe_2_NC nanozyme, a multi‐enzyme cascade antioxidant system was constructed by encapsulating Fe_2_NC in a selenium‐containing MOF (Se‐MOF) shell layer.^[^ ^53^ ^]^ Zhao et al. developed Ce‐MOF‐818 with exceptional organophosphate hydrolase‐like and catechol oxidase‐like activity.^[^ ^54^ ^]^ The close integration of Cu and Ce active sites in Ce‐MOF‐818 effectively reduced the diffusion distance of intermediates, resulting in enhanced cascade catalysis efficiency. Tang et al. designed a sulfur‐doped bimetallic single‐atom nanozyme (S‐FeCo‐NC) by anchoring cobalt atoms onto ZIF‐8@ZIF‐67 via a host–guest strategy, followed by the introduction of iron and sulfur to form atomically dispersed Fe–Co active sites.^[^ ^55^ ^]^ The resulting nanozyme exhibited laccase‐like, POD‐like, and CAT‐like activities, enabling a dual‐mode cascade sensing system for tetracycline (TC) detection. Distinct from conventional cascade nanozyme systems, Qiu et al. recently proposed a novel strategy for constructing hollow microreactors based on zeolitic imidazolate framework‐8 (ZIF‐8).^[^ ^56^ ^]^ By constructing a fluff‐like nanostructured surface via surface‐initiated living crystallization‐driven self‐assembly of micellar brushes, a high‐density catalytic layer was anchored onto the external surface of the ZIF‐8 microreactor. Meanwhile, the internal cavity was utilized to encapsulate additional functional catalysts, achieving nanoscale spatial separation and hierarchical organization of distinct catalytic sites. Such controlled organization of active sites and delicate balance of internal and external reactions effectively enhance the tandem catalytic activity.

Similarly, other hollow or multi‐hole nanomaterials have attracted significant attention in immobilized cascade catalysis owing to their unique internal cavities, large specific surface areas, and excellent mass transport properties. In this type of nanozymes, substances are transported on different active centers to form cascade reactions. For example, an efficient biomimetic dual inorganic nanozyme‐based nanoplatform was constructed by using dendritic mesoporous silica NPs as the carrier to integrate Au NPs and Fe_3_O_4_ NPs.^[^ ^57^ ^]^ Au NPs with unique GOx‐mimicking catalytic activity can catalyze glucose oxidation to produce H_2_O_2_, which is subsequently catalyzed by Fe_3_O_4_ NPs to liberate •OH for inducing tumor‐cell death. Using mesoporous polydopamine (mPDA) as the core, Zhu et al. presented a multifunctional nanoactuator by sequentially loading 2‐(phenylselanyl)ethan‐1‐amine (SePh) and MnO_2_ onto its surface through Schiff base reactions and electrostatic interactions, respectively, endowing it with glutathione peroxidase (GPx)‐like activity from SePh and SOD/CAT‐like activities from MnO_2_.^[^ ^58^ ^]^ To improve clinical applicability, biocompatible polymers are also good candidates to serve as nanocarriers to load cascade nanozymes. For example, using bovine serum albumin (BSA) nanospheres as a nanocarrier, Zeng et al. developed a biocompatible nanoplatform (BSA@Au/Pt‐IR808) by loading ultrasmall gold nanoparticles (AuNPs) and platinum nanoparticles (PtNPs).^[^ ^59^ ^]^ By integrating GOx‐like activity of AuNPs and the dual‐enzyme activity (POD and CAT) of PtNPs, the proposed nanoplatform with triple‐amplification of enzyme activity can be applied for effective combination therapy of breast cancer.

A nanozyme is essentially a nanomaterial with enzyme‐like activity. Thus, using one nanozyme as nano‐substrate for the immobilization/integration of another nanozyme is a popular approach to design cascade nanozyme.^[^ ^60^, ^61^, ^62^, ^63^, ^64^ ^]^ For example, manganese dioxide (MnO_2_) NPs with CAT‐like activity were used for loading AuNPs with GOx‐like activity to prepare the cascade nanozyme cores of MnAu.^[^ ^15^ ^]^ After coated with genetically engineered cell membranes (gCMs) overexpressing PD‐1 receptors, biomimetic nanozymes gCM@MnAu were obtained for cancer immunotherapy. Similarly, Liu and coworkers used zinc peroxide (ZnO_2_) as support to absorb peroxide‐like active platinum nanoparticles (PtNPs) to prepare self‐cascade nanozyme (denoted as ZnO_2_@Pt). ZnO_2_ released H_2_O_2_ in response to the acidic tumor microenvironment (TME).^[^ ^65^ ^]^ Yin and co‐workers used bimetallic Au–Pt NPs with triple‐enzyme‐mimicking activities to construct augmented cascade catalysis in tumor therapy. In this work, metastable Cu_2_O nanoparticles served not only as scaffolds for anchoring ultra‐small Au–Pt nanozymes, but also as a copper source for the in situ assembly of porous MOF shells, which confined and stabilized the Au–Pt nanozymes to enhance their enzyme‐mimicking activities.^[^ ^26^ ^]^ Recently,

#### 2.2.2 Nanozymes Immobilized in Microfluidics

Microfluidic systems have proven to be a powerful and promising technique in the field of biology and chemistry owing to their advantages: time‐saving, low energy consumption, rapid mass transfer, and portability.^[^ ^66^, ^67^, ^68^ ^]^ In recent years, the immobilization of nanozymes within microfluidic chips to enhance the overall efficiency, selectivity, and stability of cascade reactions has attracted considerable research interest.^[^ ^69^, ^70^ ^]^

The paper based microfluidic chip integrates the advantages of cellulosic paper, such as low cost and capillary‐driven flow, with microfluidic technology, and has emerged as a prominent platform in the microfluidic field. The emergence of bioactive paper enables the immobilization of nanozyme cascade systems on paper, facilitating the entire reaction course for quick and portable analyte detection.^[^ ^71^, ^72^, ^73^ ^]^ For example, Pang et al. constructed an all‐nanozyme cascade system by cascading CsPbBr_3_@Zr‐MOF with OXD‐like activity and Prussian blue (PB) with POD‐like activity.^[^ ^74^ ^]^ To investigate the potential of the cascade system for visual point‐of‐care testing (POCT), a portable paper‐based device was prepared for ascorbic acid (AA) analysis, with CsPbBr3@Zr‐MOF and PB‐TMB immobilized in the upper and lower holes of the device.

Polydimethylsiloxane (PDMS) and other plastic materials are commonly used in the fabrication of microfluidic chips because they permit facile formation of microchannel networks by techniques such as soft lithography. Within these polymer‐based devices, nanozymes can be immobilized by coating or surface functionalization in defined channel regions to achieve spatial separation of cascade steps. Moreover, using the unique product separation and substance flow functions of microfluidic chips, the cascade catalysis of immobilized nanozyme system is realized more quickly and effectively. For example, compared to all the previously reported bifunctional nanozymes required hours for glucose detection, Choi et al. employed nanozymes in a microfluidic device exhibited a much faster cascade catalysis (30 s) and a lower detection limit (0.8 µM).^[^ ^75^ ^]^ In addition, the sensitivity of microfluidic device showed several orders of magnitude higher than the batch reactor, likely due to the inherent features of microfluidic reactors, such as efficient mass transfer. Similarly, Zhang et al. reported a cascade nanozymatic system for AA detection using cascading N‐doped carbon nanocages and copper oxide (CuO).^[^ ^76^ ^]^ Interestingly, when the cascade catalytic reaction system was transferred from an open reactor into a spatially confined microfluidic device, the slope of the calibration curve showed nearly a 1000‐fold rise, indicating a higher sensitivity and efficiency of the reaction in the microfluidic device.

Given the characteristics of multi‐channel simultaneous detection of microfluidic chips, some studies have employed the combination of cascade nanozymes and microfluidic chips to realize the high‐throughput and multi‐modal detection of analytes. For example, Li et al. constructed a high‐throughput microfluidic device for instant POCT of circulating tumor cells (CTCs).^[^ ^77^ ^]^ Norepinephrine (NE) modified Fe_3_O_4_@SiO_2_ was used for the capture of rare CTCs. Meanwhile, Au@CuMOF‐DOTA with multienzyme mimetic activities can be used not only as a capture probe but also as a signal probe by catalyzing glucose reduction for colorimetric. Under the influence of a magnetic field, target analytes were precisely trapped at the bottom of the separation channel, enabling the precise capture and separation of CTCs. The microfluidic array chip could detect up to six samples simultaneously, which reduced reaction time and improved detection efficiency. Wei et al. developed a dual‐channel photoelectrochemical (PEC) microfluidic immunosensor by integrating AgBr‐sensitized La‐doped BiOBr with surface oxygen vacancies (AgBr/La‐BiOBr‐OV) heterojunctions and cascade catalytic iron‐phosphate‐based (FePOs) nanozymes to achieve the simultaneous and highly sensitive detection of carbohydrate antigen 15‐3 (CA15‐3) and cancer antigen 125 (CA125).^[^ ^78^ ^]^ The incorporation of La and oxygen vacancies into BiOBr significantly improved charge separation and light‐harvesting efficiency, while the FePO nanozymes, exhibiting SOD‐ and CAT‐like activities, enabled efficient oxygen regeneration to amplify the PEC signal. The developed system demonstrated the potential of combining microfluidic platforms with nanozyme cascades for multiplexed, high‐throughput clinical biomarker analysis.

#### 2.2.3 Cascade Nanozyme Encapsulated in Artificial Organelle

Inspired by living organisms, nanozyme‐based artificial organelles have attracted widespread attention from researchers in the field of biomedicine. In nanozyme cascade platforms, encapsulating nanozymes within a membrane structure confines their catalytic activity to designated regions, which helps prevent interference with native cellular pathways and simultaneously improves their biocompatibility, enabling safer and more targeted intracellular applications. Therefore, the cell membrane has been extensively utilized in nanozyme cascade systems owing to its dual advantages of promoting targeted delivery and minimizing immune response.^[^ ^79^, ^80^, ^81^, ^82^, ^83^, ^84^ ^]^ For instance, Gao reported a tumor cell membrane‐coated liposomal nanozyme platform (Ru@ATO‐Lip/M),^[^ ^85^ ^]^ in which ruthenium nanoparticles (Ru NPs) and atovaquone (ATO) were co‐loaded into liposomes and subsequently cloaked with hybrid tumor cell membranes. Ru NPs showed dual catalytic activities (GOx‐like and POD‐like) enabling sequential glucose oxidation to H_2_O_2_ and subsequent Fenton‐like generation of •OH for chemodynamic therapy. Simultaneously, ATO suppressed mitochondrial respiration, amplifying starvation effects and relieving hypoxia to sustain catalytic efficiency, thereby achieving synergistic tumor therapy. Zhao et al. recently developed a macrophage membrane‐coated multifunctional nanoreactor (CWHM) by integrating Cu‐doped WO_3–x_ (Cu–WO_3–x_) with responsive dyes.^[^ ^86^ ^]^ The Cu–WO_3–x_ component exhibited enzyme‐mimicking properties, catalyzing the breakdown of excessive H_2_O_2_ and O_2_^•−^ in inflamed tissues, thereby mitigating oxidative stress associated with hepatic injury.

On the other hand, artificial polymersomes provide improved stability, making them attractive alternatives for constructing robust bionic cascade systems.^[^ ^87^, ^88^, ^89^ ^]^ For instance, Zhang et al. established a new microcompartment by using monolayer cross‐linked zwitterionic vesicles (cZVs) with a carboxylic acid saturated cavity.^[^ ^90^ ^]^ The cascade nanozymes (cerium oxide (CeO_2_) and Pt NPs) were synthesized in the cavity of cZVs through the affinity binding of metal ions with the abundant carboxylic acid groups. The monolayer structure endows cZVs with intrinsic permeability, and O_2_^•−^ could enter the cavity easily. CeO_2_ with unique SOD‐mimicking catalytic activity can catalyze O_2_^•−^ oxidation into H_2_O_2_, while the as produced H_2_O_2_ is subsequently catalyzed by Pt NPs to transformed to H_2_O and O_2_. In vitro and vivo experiments both confirmed that the resulting artificial organelles successfully mimic peroxisome for detoxification of ROS. Recently, Xu et al. developed a biomimetic polymersome‐based system (CeO_2_@M) by assembling cerium dioxide nanoparticles with a negatively charged lipid membrane to mimic endothelial cell functions during the early phase of tissue‐engineered vascular graft (TEVG) transplantation.^[^ ^91^ ^]^ The system exhibited multienzymatic nanozyme activity, including apyrase‐like, SOD‐like, POD‐like, and CAT‐like functions, enabling the degradation of ATP/ADP and reactive oxygen species. This enzymatic cascade not only reduced platelet aggregation and blood cell adhesion on the graft surface but also promoted vascular remodeling by stabilizing the local biochemical microenvironment. The work highlights the potential of polymersome‐encapsulated nanozymes for regulating immune responses and enhancing graft integration in tissue engineering applications.

#### 2.2.4 Cascade Nanozyme Catalytic System Localized by Bridging DNA

Due to its structural programmability, reaction predictability, and excellent specificity and sensitivity, DNA building blocks have shown great potential in the regulation of nanozyme cascade system. Researchers have developed localized cascade catalytic systems using bridged DNA probes, which effectively enhance catalytic efficiency and have been further leveraged to construct a variety of sensitive and efficient biosensing strategies. For example, Gong et al. synthesized two nanozymes with GOx‐like and POD‐like activities, respectively, by controlling AuNPs with tunable exposed facets immobilized on the ZIF‐8 dodecahedrons.^[^ ^92^ ^]^ Based on these two nanozymes, a cascade system was spontaneously generated through the target bridging via base‐complementary reaction, and realized colorimetric/photoelectrochemical (PEC) dual‐mode antibiotic resistance genes (ARGs) detection. In Yuan's work, a novel nanozyme cascade platform combined with Mn_3_O_4_@AuNPs nanozyme with GOx‐like activity and hemin/G‐quadruplex DNAzyme with POD‐like activity was successfully constructed to detect microRNA‐499.^[^ ^93^ ^]^ Notably, DNA origami and related techniques can be employed to achieve the directional assembly of distinct nanoparticles, thereby ensuring the sequential order and catalytic efficiency of substrates, which holds significant potential for therapeutic applications. For example, Ding et al. described a DNA origami‐based enzymatic cascade nanoreactor (DOECN) by integrating AuNPs and ferric oxide (Fe_2_O_3_).^[^ ^94^ ^]^ Leveraging the GOx‐mimicking activity of AuNPs and the POD‐like activity of Fe_2_O_3_, the DOECN facilitates H_2_O_2_ production, glutathione (GSH) depletion, and intracellular acidification in a synergistic manner. These processes collectively enhanced Fenton‐like reactions and promoted the generation of ROS, particularly •OH, thereby significantly improving the efficacy of chemodynamic therapy (CDT).

## 3 Physicochemical Properties Influencing Nanozyme Cascade Reaction

**Figure 3:**
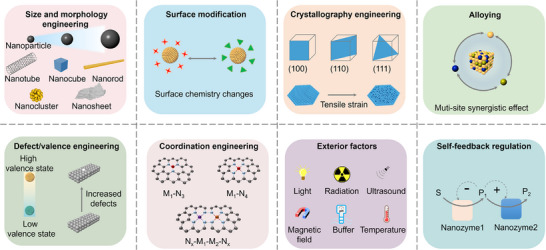
Strategies for influencing catalysis selectivity and activity toward cascade nanozyme. Reproduced with permission.^[^ ^95^ ^]^ Copyright 2022, Wiley‐VCH.

All‐nanozyme cascade systems have garnered widespread attention in recent years for their ability to achieve multistep catalytic conversion without relying on exogenous nature enzymes. Compared with a single catalytic process, a cascade reaction can efficiently channel substrates and sequentially convert them into final products by integrating multiple enzyme‐like activities at the nanoscale. However, the catalytic mechanism and rational design of cascade nanozymes are also more complex and elusive. Therefore, there is an urgent need for precise and multiscale design from material atom/surface scale to macroscopic configuration and environmental regulation to achieve controllable multi‐stage catalytic behavior. Guided by these objectives and governing factors, the common strategies can be summarized as follows: size/morphology engineering, surface modification, crystallography engineering, alloying, defect/valence engineering, coordination engineering, microenvironment and external field control, and self‐feedback regulation (**Figure** **3**).

In the following sections, we delineate the physicochemical essence of these dominant mechanisms and highlight their effects on reaction‐pathway selection and efficiency. We also discuss representative examples of classic strategies and highlight how these strategies improve the overall efficiency of cascade catalysis by distributing active sites, reducing key activation barriers, and enhancing site selectivity. It should be emphasized that the catalytic performance of cascade nanozymes may result from the combined action of multiple mechanisms. For example, in addition to the exposure of active sites, the adjustment of size and morphology can sometimes cause lattice distortion and active facets change. Consequently, the classification adopted in this review is based on the dominant or most distinctive mechanism, rather than a strict mutually exclusive division. We aim to provide readers with a design framework based on causal logic to identify that facilitates the identification and integration of different regulatory strategies in future design of cascade nanozymes.

### 3.1 Size and Morphology Engineering

The size and morphology of the nanozyme are key parameters to adjust the overall performance of cascade catalysis. On the one hand, these structural parameters control the exposure of the active site, thereby directly affecting adsorption and desorption as well as the kinetics of intermediate transport. On the other hand, the transformation of size and shape would also induce scale‐dependent electronic structure adjustment, which in turn modulates reaction energy barriers. Accordingly, size and morphology engineering is not only a means for increasing active site utilization, but also an effective strategy for optimizing cascade reaction pathways and kinetics.

Size modulation induces changes in specific surface area, fraction of low‐coordination surface atoms and local electronic structure, which produce pronounced size‐dependent catalytic behavior.^[^ ^96^ ^]^ For example, the SOD‐mimicking activity of CeO_2_ is typically observed only for small‐sized nanoparticles (less than 5 nm). It may be probably because smaller particles generate additional surface oxygen vacancies that help maintain a high Ce^3+^ fraction.^[^ ^97^, ^98^ ^]^ Similar trends have also been reported for noble‐metal nanoparticles. Li et al. systematically demonstrated a strong dependence of the antioxidant enzyme‐like activities of RuNPs on particle size.^[^ ^99^ ^]^ By comparing RuNPs of 2.0, 3.9, and 5.9 nm, they found that reducing particle size enhanced both CAT‐like and SOD‐like activities. Mechanistic investigations indicated that this enhancement arose from the larger specific surface area and higher ratio of oxidized Ru associated with smaller sizes, which increased the density of reactive sites and decreased energy barrier of key reaction steps. Similarly, Zhang et al. employed atomic layer deposition (ALD) to regulate the size of PtNPs (0.55–2.81 nm), generating a series of Pt/CNTs nanozymes.^[^ ^100^ ^]^ After systematically characterizing their electronic and kinetic effects in the antibacterial process, a volcano‐type dependence of intrinsic antibacterial activity on size was revealed. Mechanistic analysis indicates that an appropriate nanoparticle size both preserved a relatively high surface Pt^0^/Pt^2+^ ratio and achieved a balance between substrate adsorption and desorption, which was conducive to realize the excellent catalytic performance and antibacterial activity.

Morphological engineering affords an additional degree of catalytic activity regulation strategy. Classically, by transforming nanomaterials from solid to hollow and from dense to porous, the surface‐to‐volume ratio is increased, optical and thermal responsiveness are introduced, and diffusion and mass‐transport pathways are reconfigured. For example, Ye et al. found that proton‐induced metal replacement during acid etching transformed CoFe Prussian blue (CFPB) nanocubes into CFPB nanoframes with emergent Fe^3+^─N≡C─Fe^2+^ motifs and high surface area.^[^ ^101^ ^]^ Unlike Prussian blue nanoparticle as a ROS‐scavenger, both the CFPB nanocubes and nanoframes can catalyze the water‐initiated tandem reaction (H_2_O → O_2 _→ H_2_O_2_ → •OH), and nanoframes produced the highest •OH yield. Density functional theory (DFT) calculations indicated that spatially separated active sites in CFPB enabled water oxidation, oxygen reduction and Fenton‐like reactions. In another study, researchers also found that nanozyme engineered porous or channelled architectures could accelerate mass transport and maximally exposed active sites during reaction.^[^ ^102^ ^]^

### 3.2 Surface Modification

Given the nature of nanozymes as heterogeneous catalysts, their interfacial properties and structural characteristics play a pivotal role in dictating catalytic performance and enzyme‐like activity types. Altering surface charge is a widely employed strategy in surface modification to regulate nanozyme activity. Gao et al. proposed a surface engineering strategy by using polystyrene sulfonate (PSS) to improve the specific activity of Ru nanozymes.^[^ ^103^ ^]^ After coating with PSS, charge transfer occurred from Ru to the coated ligand. The charge transfer induced by PSS ligand modification made Ru become less electron‐rich, resulting in weaker •OH affinity and higher POD‐like activity. In Jiang's report, AuNPs modified with amine ligands exhibited higher catalytic activity than those with other ligands or without ligands.^[^ ^104^ ^]^ It may be attributed to polyamine ligands, whose higher density of amine electron donors on the AuNP surface promoted ROS conversion. Therefore, ligand exchange can modulate AuNPs between active and inactive states.

In the case of nanozymes that require ligand‐assisted synthesis, surface ligands not only act as dispersing or stabilizing agents but also serve as spatial guides and substrate‐binding modules. By coupling different active sites, they transform nanoparticles into “nano‐reactors” that enable the ordered transformation and delivery of substrates on a single nanozyme. A representative example is polyadenine (polyA)‐stabilized AuNPs, which simultaneously exhibit GOx activity (producing H_2_O_2_) and H_2_O_2_‐dependent POD‐like activity.^[^ ^105^ ^]^ The combination creates a cascade reaction system within a single nanoparticle. Shen et al. reported a class of DNA‐functionalized AuNP nanozymes, demonstrating that DNA functioned not only as a stabilizer but also as the scaffold for catalytic transformation.^[^ ^106^ ^]^ Mechanistic studies revealed that the long‐range oxidation reaction starts with the homolytic cleavage of H_2_O_2_ on the AuNP surface to generate radicals. These radicals migrated along the DNA corona and completed oxidation at substrate‐binding sites within the DNA bases. Therefore, the AuNP core served as the radical generation center, while the DNA corona provided both a radical transporter and substrate recognition sites. This strategy improves catalytic efficiency and confers substrate selectivity of nanozyme, highlighting the potential value of surface modification engineering as a strategy for designing substrate‐specificity catalysts.

### 3.3 Crystallography Engineering

Crystallographic engineering reshapes the interfacial interactions and electron transfer of nanozymes, thereby fundamentally regulating the adsorption, activation, and release of substrates and intermediates at the catalytic sites. For example, the lattice strain caused by stretching or thermal treatment can effectively adjust the d‐band center and local electronic density of metals.^[^ ^107^, ^108^, ^109^ ^]^ This modulation alters substrate adsorption energies, transition‐state barriers, and electron transfer dynamics, thereby enabling cooperative enhancement or selective amplification of multiple enzyme‐mimicking activities within one nanostructure. As a typical example, Li et al. modulated the electronic state of Ru‐based nanozymes by facilely controlling the lattice spacing of Ru nanocrystals under various calcination temperatures.^[^ ^110^ ^]^ Moreover, the corresponding electronic modulation improved the response ability of Ru nanoparticles to external electric fields. DFT calculations revealed that lattice expansion and electrical stimulation enhanced multiple enzyme‐like activities by reducing the electron density and shifting the d‐band center of Ru active sites. This work opens up horizons for catalytic optimization of nanozymes. Another example was reported by Liu's group.^[^ ^95^ ^]^ Lattice tensile strain was introduced into palladium nanosheets (Pd NSs) via surface reconstruction. The resulting lattice strain led to the lengthening of Pd─Pd bond lengths and many structural defects, thereby activating the otherwise inert (111) facet. DFT calculations revealed that tensile strain promotes the photodynamic and enzyme‐like activities (CAT‐like, POD‐like). Compared with unstrained Pd nanosheets, the strained Pd NSs (SPd NSs) showed approximately five times higher tumor inhibition rate. Recently, Chong et al. discovered that hydrogen incorporation into palladium nanomaterials can tune the structural and electronic properties of Pd nanocrystals, enabling precise modulation of their enzyme‐like catalytic activity and substrate selectivity.^[^ ^111^ ^]^ Pd/H_2_ nanocubes (Pd NCs), which are synthesized by directly injecting hydrogen gas into a solution containing Pd NCs, exhibit a selective enhancement in antioxidative activity against cytotoxic H_2_O_2_, O_2_^•−^, and •OH due to the sustained release of bioreductive hydrogen. In contrast, stable Pd hydride NCs (PdH NCs), which are prepared through the in situ catalytic decomposition of alternative sources of hydrogen atoms, exhibit a remarkable enhancement in exclusive H_2_O_2_ activation pathways, specifically exhibiting POD‐like and CA)‐like activities. Spectroscopic characterization combined with DFT calculations revealed that this high catalytic activity and specificity of PdH NCs arise from lattice tensile strain and electronic structure change. This study not only demonstrated that hydride formation can affect both the activity and selectivity of Pd nanozymes but also provided a viable strategy for the precise regulation of specific enzyme‐like activity in hydrogen‐loading nanozymes.

### 3.4 Alloying

Alloying provides an effective strategy to couple multiple enzyme‐like activities. By precisely tuning the metal composition at the nanoscale, distinct catalytic sites with complementary activities can be engineered to create a cascade reaction. For example, Lin et al. developed a trimetallic alloy nanozyme (designated as ACPP) with five enzyme‐like activities.^[^ ^112^ ^]^ DFT calculations revealed that the metal active sites acting in each step of each enzymatic reaction were different, and there were synergistic effects among them, which could greatly improve the catalytic efficiency. Briefly, the role of the Cu and Pt sites facilitates the adsorption, activation, and dissociation of H_2_O_2_ and HO_2_•, which are the preferred binding sites for the reaction. In addition, the Cu sites favor further deprotonation of OH* to form O*, and the Au site was conducive to the desorption of H_2_O_2_, •OH, and O_2_. This reflected the synergistic effect of each element of the AuCuPt nanozymes, which jointly determined the excellent enzyme‐like catalytic. Such a division of labor substantially lowered the energy barriers of each reaction step, thereby achieving higher catalytic efficiency and multienzyme‐induced enzymatic cascade catalysis.

Compared to a single metal nanozyme, the alloy nanozyme displayed superior catalytic performance and revealed the unique active sites‐separated characteristic, thus giving the excellent reactive specificity to proceed with tandem or parallel reactions. A representative example is a bimetallic ruthenium–cobalt (RuCo) nanosheet reported by Shi et al.^[^ ^113^ ^]^ DFT calculations indicate that the catalytic performance of the ruthenium–cobalt bimetallic composition surpasses that of its single‐component counterpart, revealing SOD‐like and CAT‐like catalytic reaction processes on different crystal facets. Specifically, the RuCo (011) facet of RuCo nanosheets exhibited pronounced CAT‐like activity, while the (002) crystal facet exhibits superior SOD‐CAT‐like cascade enzymatic catalytic performance compared to the (002) crystal facets of Ru and Co. The alloy structure of RuCo nanosheets induces ensemble effects, thereby enhancing the catalyst's performance. Recently, Fan et al. developed nanozymes possessing both SOD‐ and MPO‐like activities by adjusting the alloy ratio.^[^ ^114^ ^]^ Among a series of ultrasmall metal nanozymes: Au, Au_3_Pd_1_, Au_2_Pd_2_, Au_1_Pd_3_, Pd, they find that AuPd alloy nanozyme exhibits the highest cascade activity when the ratio of Au and Pd is 1:3. DFT calculations revealed that fine control of the Au:Pd ratio induced an upward shift of the d‐band center and electronic state modulation, markedly enhancing the adsorption and activation of superoxide anions. This work demonstrated that nanozyme‐based systems can employ alloying as a strategy for structural and electronic modulation, enabling precise control and optimization of catalytic performance.

### 3.5 Defect and Valence Engineering

Defect engineering has emerged as a highly effective approach for enhancing the catalytic activity.^[^ ^115^, ^116^, ^117^ ^]^ For example, Liu et al. demonstrated that nitrogen vacancies generated through C‐N covalent coupling were the pivotal factor in enhancing the POD‐like performance of VN/rGO composites.^[^ ^118^ ^]^ By enriching the electronic structure, stabilizing defective sites, and lowering the catalytic energy barrier, the material achieved activity far beyond that of natural enzymes. This work offers a novel pathway for designing efficient and durable nanozymes by integrating strong interfacial coupling with defect engineering.

Valence engineering refers to techniques that improve material properties by manipulating the valence states of its elements. In recent research, valence engineering is widely used in the study of nanozymes. For example, the multienzyme‐like functionality of Mn_x_O_y_ nanozyme depends on the synergistic regulation between reversible redox cycling of the mixed valence state and a high density of surface oxygen vacancies.^[^ ^119^, ^120^ ^]^ Moreover, valence engineering of other transition metal oxides yields parallel behavior. For instance, a mild oxidation valence‐engineering strategy applied to MoO_3‐x_ was used to shift the average molybdenum valence of Mo from 4.64 to 5.68.^[^ ^121^ ^]^ Experimentally, the low valence MoO_3‐x_ sample with average valence 4.64 exhibited predominantly CAT‐like activity, converting H_2_O_2_ to O_2_, whereas the high valence sample with average valence 5.68 displayed predominantly POD‐like activity, decomposing H_2_O_2_ to form hydroxyl radical.

As described above, defect engineering modulates electron density and intermediate binding by adjusting vacancy types and concentrations; valence engineering tunes redox driving forces and reaction pathways by adjusting the average oxidation states of metal centers. When these approaches are combined, the resulting coupling between defect sites and metal valence can provide synergistic control over charge transfer, intermediate adsorption and desorption, and the energy barrier of rate‐determining steps. Therefore, coupling of two strategies offers a practical route to reprogram catalytic selectivity and to endow nanozyme with multi‐enzyme activity. A representative example is CeO_2_ nanozymes, which exhibit diverse redox‐mimetic activities owing to their tunable Ce^3+^/Ce^4+^ redox couple and abundant surface oxygen vacancies. According to abundant mechanistic studies, CAT‐like activity is predominantly initiated at surface Ce^4+^ sites and mediates the decomposition of H_2_O_2_ into O_2_ and H_2_O, and SOD‐like activity arises from electron transfer between Ce^3+^ and Ce^4+^, enabling the dismutation of O_2_^•−^.^[^ ^122^, ^123^, ^124^, ^125^ ^]^ By contrast, POD‐like activity is closely associated with surface Ce^3+^ species and oxygen vacancies, which facilitate H_2_O_2_ activation and the generation of highly reactive intermediates that oxidize substrates.^[^ ^126^, ^127^, ^128^ ^]^ Taken together, electronic shuttling between mixed valence states and the coupling among differently valent active sites permit a single CeO_2_ particle to carry out sequential cascade conversions, for example O_2_^•−^ to H_2_O_2_ to H_2_O.

### 3.6 Coordination Engineering

The single‐atom nanozymes (SAzymes) provide an ideal platform for fine‐tuning catalytic activities at the atomic scale, because of their well‐defined geometric and atomically dispersed catalytic sites. Coordination engineering constitutes a central strategy for the rational modulation of SAzymes catalytic performance. In essence, alterations to the first and second coordination shells of a metal active site (including ligand identity, coordination number, and proximal heteroatoms) induce local electronic reorganization and shifts in the d‐band center, thereby tuning the adsorption‐activation‐desorption kinetics of substrates and intermediates. For example, Zhang et al. introduced B atoms into the second coordination shell of Zn atoms during the pyrolysis of the zeolite‐like Zn‐based boron imidazolate framework.^[^ ^129^ ^]^ The formed Zn‐N‐B bonds resulted in an upward shift of the d‐band center (E_d_) of Zn toward the Fermi level (E_f_) and improved the oxidation state of Zn by facilitating the electron transfer from Zn to N to B. The charge redistribution improved H_2_O_2_ and O_2_ adsorption capacity of the nanozymes and nanozyme‐substrate electron transfer, dramatically boosting the multi‐enzyme‐like activities (POD, CAT, and OXD) of Zn‐SAs@BNC_1000_. Analogously, Zhan et al. prepared two single‐atom carbon dots with CuO_4_ coordination environment (Cu‐SLCDs) and CuN_4_ coordination environment (CuN‐CDs).^[^ ^130^ ^]^ Both nanozymes exhibit multienzyme‐like (OXD, POD, SOD, CAT) activities, with Cu‐SLCDs showing higher activity levels. Experiments and theoretical calculations demonstrate that, compared to CuN‐CDs, the polyphenol p‐π conjugated structure creates a unique electron‐donating coordination environment, which increases the electron density of Cu‐SLCDs at the Fermi level, enhances electron transfer, and boosts multienzyme‐like activities. This work highlights the potential of ligand design, particularly π‐conjugated natural polymers, to improve multienzyme activity.

Compared with single‐atom nanozymes, dual‐atom architecture offers a more direct approach for simulating the multi‐site synergistic mechanisms of natural enzymes to achieve outstanding catalytic activity.^[^ ^131^ ^]^ For example, Guo et al. constructed a Fe─Mn dual‐atom nanozymes (DAzymes) on edge‐rich N‐doped porous carbon (Fe_1_Mn_1_─NC_e_) with excellent POD‐like and OXD‐like performance.^[^ ^132^ ^]^ DFT and experimental analyses revealed that the introduction of Mn‐adjacent sites leads to the clustering of trace electrons on the Fe sites and drives the d‐band center of the Fe sites upward closer to the Fermi energy level, resulting in the enhancement of adsorption capacity for the substrate, accelerated dissociation of H_2_O_2_, weakening of the O─O bonds on the O_2_, and balancing the free energy of the O* intermediate. Consequently, the optimized H_2_O_2_/O_2_ adsorption and activation capabilities enable Fe_1_Mn_1_‐NC_e_ to exhibit a favorable catalytic path, explaining its high multiple‐enzyme‐like activity. Thus, deliberate placement of a second metal near a primary single‐atom site is an effective design principle for increasing substrate affinity and multi‐enzyme activity. It is important to emphasize that coordination engineering is not merely the simplistic addition of a heteroatom. In fact, it entails multi‐level, atom‐scale adjustments (first coordination shell, second coordination shell, support edge structure, π‐conjugated ligands, etc.) that jointly alter both electronic states and geometric constraints, thereby optimizing thermodynamics and kinetics of catalytic pathways. For example, when a Fe─N_4_ single‐atom site is placed near a double‐atom or heterometallic site, it can preserve atomic dispersion and create new adsorption configurations or reduce desorption barriers for intermediates, thereby enabling fine control over multi‐enzyme selectivity.^[^ ^133^ ^]^

### 3.7 Exterior Factors

In addition to modulating the intrinsic activity of catalytic sites via material composition and structural design, microenvironment engineering and external‐field actuation are two important exterior factors for precise regulation of nanozyme catalysis.^[^ ^110^, ^134^, ^135^, ^136^, ^137^, ^138^ ^]^ Taking microenvironmental response as an example, recent work on sulfur quantum dots (SQDs) revealed that pH‐dependent adsorption and electronic‐level alignment determine the selection of cascade path.^[^ ^139^ ^]^ Mechanistic investigations revealed that the adsorption energy and electron level matching of •OOH and H_2_O_2_ on SQD surface are significantly different under different acid and base conditions. Under acidic conditions, the generated H_2_O_2_ undergoes a homolytic reaction to generate •OH and OH* intermediates, corresponding to the sequence of SOD‐POD. Under alkaline conditions, H_2_O_2_ preferentially undergoes disproportionation to form H_2_O and O_2_, corresponding to the sequence of SOD‐CAT. The difference in energy barriers between these pathways provides the physicochemical basis for switching the cascade route. Exploiting this microenvironmental sensitivity, it is possible to purposefully alter local chemical conditions to selectively enhance or suppress specific cascade steps, thereby functionally emulating or substituting the local regulatory strategies of natural enzymes. Microenvironmental engineering can also modulate the reaction conditions of the pore or interface through the local proton/ion release, so as to solve pH mismatch of different cascade steps. In Wei et al.’s study, poly(acrylic acid) (PAA) was confined into the channels of PCN‐222‐Fe nanozyme.^[^ ^140^ ^]^ The carboxyl group of PAA released protons in the pore cavity, which reduced the microenvironmental pH of channels and was more conducive to POD‐like activity.

External field‐driven strategies have been used to engage electron dynamics directly by providing additional energy channels, thereby enabling amplification or modulation of cascade reactions at the kinetic level.^[^ ^75^, ^141^ ^]^ For example, a chiral plasmonic nanoparticle system composed of D‐GSH‐AuNPs (D‐Au) and palladium‐coated L‐GSH‐AuNPs (L‐AuPd) was reported by Kim et al.^[^ ^142^ ^]^ in which right‐handed and left‐handed circularly polarized light (CPL) were applied alternately to produce a sequential enhancement of GOx‐like and POD‐like activities. Under right‐handed CP illumination, D‐glucose molecules were selectively bound at the D‐Au active site and transformed into gluconic acid and H_2_O_2_. Under subsequent left‐handed CP illumination, the generated H_2_O_2_ was decomposed into •OH at the L‐AuPd active site. By controlling the kinetics of electron transfer, the chiral system provided a marked increase in overall catalytic efficiency compared to non‐controlled cascade reactions. This work offers a promising approach for precise regulation of the cascade pathway by means of external light fields. Additionally, high‐energy external fields such as X‐rays have also been applied to enhance valence‐state cycling and catalytic activity at single‐atom sites. Zhang et al. reported an atom‐level engineered FeN_4_‐centered nanozyme (FeN_4_‐SAzyme), which exhibited an external‐field‐enhanced enzyme‐like activities.^[^ ^143^ ^]^ The resulting FeN_4_‐SAzyme showed POD‐like and GSHOx‐like activities and was capable of triggering a single‐site self‐cascade enzymatic reaction to convert intracellular H_2_O_2_ to produce •OH and deplete GSH. Moreover, the introduction of X‐rays promoted the conversion frequency of Fe^II^/Fe^III^, thereby remarkably enhancing such self‐cascade enzymatic activity. These results demonstrated the ability of the external field to amplify cascade reactions by accelerating critical electron‐transfer processes.

Combining external stimuli with the local microenvironment has further expanded the paradigm of controllable cascade reactions. For example, Liu et al. reported a guanine‐derived single‐atom copper nanosheet (G–Cu), which exhibits the catalytic cascade property of GOx and POD.^[^ ^144^ ^]^ Photoexcitation of semiconductors produced the electron (e^−^) and positive hole (h^+^) pairs. Then, the photo‐generated positive hole (h^+^) oxidized glucose to form gluconic acid, thereby rapidly reducing the pH of the local microenvironment. At the same time, photo‐generated electron (e^−^) would reduce O_2_ to H_2_O_2_ in the acid environment provided by proton and gluconic acid. Finally, the atomically dispersed Cu─N coordination sites of G─Cu preferentially catalyzed H_2_O_2_ decomposition to produce •OH and O_2_^•−^ under the resulting acidic conditions. From the perspective of a design‐driven approach, the integration of microenvironment engineering with externally applied stimuli can offer a clear design idea for the rational construction of programmable, controllable and highly efficient nanozyme cascade systems.

### 3.8 Self‐Feedback Regulation

Self‐feedback regulation offers a novel method for the rational design of cascade reactions, which is different from the conventional passive control. By exploiting feedback generated from reaction intermediates or products, these systems can autonomously tune their catalytic activity, thereby achieving spatiotemporal control over the outputs of cascade reactions. Unlike the allosteric modulation observed in natural enzymes, nanozymes typically lack reversible conformational transitions. Nevertheless, the self‐feedback regulation may be engineered through product‐structure coupling, release of active species, and in situ material reconstruction. These strategies amplify, inhibit or terminate catalytic processes and thus limit excessive conversion and reduce undesired side reactions. A representative example was reported by Fan'group.^[^ ^145^ ^]^ During glucose oxidation, AuNPs generated H_2_O_2_, which in turn induced the AuNPs’ seeded growth in the presence of chloroauric acid (HAuCl_4_). In this system, the concurrent regulation of AuNP size, morphology, and catalytic activity was controlled by two intrinsic negative feedback mechanisms: the decline in catalytic efficiency with increasing particle size and the surface passivation induced by the reaction product, gluconic acid. Although this work was an early example of its kind, it provided direct evidence for a “product→structure→activity” feedback loop and supports product‐driven self‐regulation strategies. This work demonstrated that catalytic products could not merely be outputs but also serve as feedback signals that altered the physicochemical properties of nanomaterials and thereby modulated catalytic kinetics and selectivity.

In more complex cascade reactions, the construction of feedback regulation can be used both for feedback to avoid overcatalysis and as feedforward to facilitate downstream reactions. Zhang et al. developed a cascade nanozymatic network with feedback and feedforward functions by exploiting Co─N‐doped carbon nanotubes (Co‐N‐CNTs), in which different substrates compete at the same active sites.^[^ ^70^ ^]^ In this system, intermediate H_2_O_2_ competitively occupied Co─N_4_ sites and thereby inhibited the first one‐electron oxidation of TMB, producing negative feedback that limited the rate of the first step. At the same time, the generated H_2_O_2_ served as a strong oxidant that accelerated the micellar nanozyme‐catalyzed second one‐electron oxidation in the downstream step, producing a positive feedforward effect on the cascade reaction catalysis. Regulation based on competitive adsorption of intermediates and active‐site occupation demonstrated the feasibility of intrinsic interaction‐driven self‐regulation in nanozyme cascade systems. Design strategies in some recent reports have extended this concept. For example, chemical gradients are used to induce in situ synthesis or activation of nanozymes, thereby avoiding excessive catalysis and toxic side reactions.^[^ ^146^, ^147^, ^148^ ^]^ Although systematic reports of self‐regulating nanozyme systems remain relatively scarce, the self‐feedback design offers valuable implications for building intelligent catalytic systems.

## 4 Validation Methods for ROS in Cascade Nanozyme Systems

ROS, such as •OH, O_2_^•−^, and ^1^O_2_, play pivotal roles in the catalytic process of cascade nanozymes. Precise identification of these species is essential for mechanistic elucidation and rational nanozyme design. However, due to their extremely short lifetimes and high reactivity, the direct detection of ROS remains a considerable challenge. To ensure reliable interpretation, in most research reports, there are usually three complementary recognized strategies: electron spin resonance (ESR) spectroscopy, free radical scavenging (quenching) assay, and the use of specific probes. Each method has its own distinct advantages: ESR provides direct spectroscopic evidence, the free radical scavenging assays provides mechanistic validation through inhibition, and the selective probe method enables real‐time visualization. These approaches work together to form a comprehensive framework for verifying the generation of ROS in nanozyme‐catalyzed cascade systems and identifying ROS types.

### 4.1 Electron Spin Resonance Spectroscopy

ESR is currently the most widely used and reliable technique for detecting short‐lived ROS, which are generated in nanozyme‐catalyzed reactions. In this method, spin‐trapping agents are used to capture transient free radicals, forming a stable free radical adduct. These adducts can be detected by ESR. As has been reported,^[^ ^47^, ^149^ ^]^ 5,5‐dimethyl‐1‐pyrroline N‐oxide (DMPO) can act as an effective trapping agent for •OH and O_2_^•−^, and 2,2,6,6‐tetramethylpiperidine (TEMP) is a specific spin trap agent for ^1^O_2_. In the ESR spectrum, the •OH‐DMPO spin adduct exhibits a typical intensity ratio of peaks 1:2:2:1 in aqueous solution signaling •OH. The 1:1:1:1 quartet pattern observed in methanol corresponds to the O_2_^•−^‐DMPO spin adduct. The spectrum of ^1^O_2_‐TEMP adduct exhibits a distinct triplet with equal intensities. Thus, ESR spectra can provide clear evidence for free radical generation and serve as a principal technique in elucidating ROS mechanisms of nanozyme cascade reaction.

### 4.2 Free Radical Scavenger (Quenching) Assay

In addition to ESR analysis, free‐radical scavenging experiments offer an indirect but effective method for confirming specific ROS species. In this method, selective scavengers are used to quench free radicals generated during the reaction. The final determination is made by monitoring the resulting changes in catalytic activity or product formation. For example, common scavengers of •OH radical include *tert*‐butyl alcohol^[^ ^150^ ^]^ and isopropanol,^[^ ^118^, ^134^, ^151^, ^152^ ^]^ O_2_^•−^ radical can be selectively removed by *p*‐benzoquinone^[^ ^118^, ^134^, ^150^, ^151^ ^]^ or superoxide dismutase,^[^ ^152^ ^]^ while ^1^O_2_ is quenched by agents such as TEMP,^[^ ^150^ ^]^ potassium iodide,^[^ ^151^ ^]^ sodium azide,^[^ ^118^ ^]^ triphenylamine,^[^ ^96^ ^]^ L‐histidine,^[^ ^152^ ^]^ or L‐tryptophan.^[^ ^134^ ^]^ Other uncommon examples include 1,1‐diphenyl‐2‐picrylhydrazyl as a broad‐spectrum ROS scavenger and catalase for H_2_O_2_ removal.^[^ ^152^ ^]^ Additional reactive oxygen species, like oxygen vacancies can be eliminated by disodium ethylenediaminetetraacetate.^[^ ^118^ ^]^ In practical application, a significant decrease in catalytic efficiency or output signal intensity of corresponding ROS‐involved reaction after addition of a specific scavenger. It should be noted that the careful design of control experiments is necessary to avoid erroneous judgement.

### 4.3 Specific Fluorescent and Chromogenic Probes

The use of selective probes is another strategy for the detection and discrimination of ROS, featuring diversity and high sensitivity. These probes exhibit characteristic fluorescence or absorbance changes after reacting with specific ROS species. After a comprehensive review of numerous literature, it was found that specific probes for •OH radical include hydroxyphenyl fluorescein,^[^ ^153^ ^]^ coumarin,^[^ ^152^ ^]^ and the salicylic acid‐dihydroxybenzoic acid chromogenic system,^[^ ^58^ ^]^ After undergoing hydroxylation reactions, these probes will generate measurable fluorescence or colorimetric signals. O_2_^•−^ radical is typically detected using dihydrorhodamine‐123 (DHR‐123) and dihydroethidium (DHE),^[^ ^149^, ^153^ ^]^ both of which become fluorescent after oxidation to oxDHR‐123 (emitting at 525 nm) and oxDHE (emitting at 630 nm), respectively; the nitroblue tetrazolium (NBT) reduction assay can also be employed for this purpose.^[^ ^152^ ^] 1^O_2_ can be monitored using 9,10‐anthracenediyl‐bis(methylene)dimalonic acid,^[^ ^153^ ^]^ whose absorbance decreases as it reacts with ^1^O_2_. H_2_O_2_ detection is often carried out by the classic HRP‐TMB colorimetric system that develops a characteristic absorption peak at 652 nm or using *p*‐hydroxyphenylacetic acid,^[^ ^152^ ^]^ analyzed via fluorescence spectroscopy (excitation at 310 nm, emission at 410 nm). Tris(4,7‐diphenyl‐1,10‐phenanthroline) ruthenium(II) dichloride often functions as the O_2_‐sensitive probe, whose intrinsic fluorescence (λ_max_ = 632 nm) undergoes oxidative quenching.^[^ ^149^ ^]^ Although such probes provide convenient and sensitive detection, some may exhibit partial cross‐reactivity with multiple ROS species; therefore, combining probe‐based analysis with ESR and scavenger experiments allows for a more accurate and comprehensive identification of ROS types.

## 5 Types of Cascade Catalytic System

**Figure 4:**
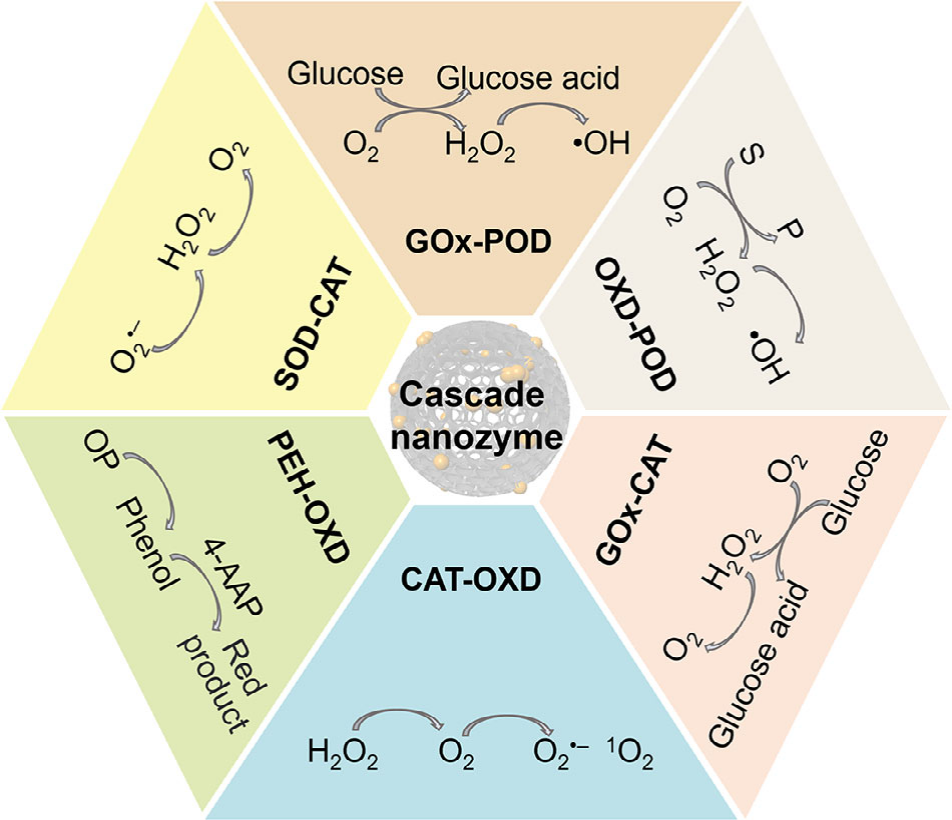
Types of classic nanozyme cascade systems.

According to enzyme‐like activities of cascade nanozymes, the reported cascade catalytic systems can be categorized into several types, including GOx‐POD, OXD‐POD, GOx‐CAT, CAT‐OXD, PEH‐OXD, SOD‐CAT (**Figure** **4**), and multi‐enzyme‐mimicking structure that integrates multiple catalytic sites within single nanoplatform. The following subsections describe the catalytic mechanisms and representative examples of each cascade type in detail, highlighting how different combinations of enzyme‐like reactions achieve synergistic functionality. To provide a comprehensive overview and facilitate comparison among different types, **Table** **1** summarizes representative all‐nanozyme cascade systems, including their material composition and architecture, cascade steps, reported catalytic parameters, assay conditions, performance metrics, and biocompatibility data.

**Table 1:** Summary of representative all‐nanozyme cascade systems.

Material composition	Cascade steps	Reported catalytic activities	Assay conditions	LODs or therapeutic efficacy metrics	Biocompatibility /toxicity	Refs.
Ir/CeO_2_ single‐atom nanoislands	GOx‐POD	GOx (glucose): V_max_ = 23.2 × 10^−8^ M s^−1^, K_m_ = 0.78 mM	pH = 7.4	100 µL of the specified solution (intravenous injection), once a week	Cytotoxicity: negligible cytotoxicity (≤ 40 ppm). Hemolysis: no significant hemolysis (Concentrations of Ce: 20–100 ppm).	[154]
		POD (H_2_O_2_): V_max_ = 23.2 × 10^−8^ M s^−1^, K_m_ = 0.06 mM				
Multienzyme cooperative hybrid nanoplatform (MSN‐Au@CO)	GOx‐POD	GOx (glucose): V_max_ = 0.01 mM min^−1^, K_m_ = 7.44 mM	not available	50 µg mL^−1^ (intragingival injection), once every 2 days	Cytotoxicity: 11.5% (50 µg mL^−1^). Hemolysis: no significant hemolysis (100 µg mL^−1^).	[155]
		POD (H_2_O_2_): V_max_ = 1.01 × 10^−6^ M min^−1^, K_m_ = 47.29 mM	pH = 4.0			
Au–Pt bimetallic aerogels	GOx‐POD	GOx (glucose): V_max_ = 3.42 × 10^−8^ M s^−1^, K_m_ = 10.47 mM	pH = 7.0, *T* = 50 °C	5 µM (glucose)	Not available.	[156]
		POD (H_2_O_2_): V_max_ = 5.04 × 10^−8^ M s^−1^, K_m_ = 19.56 mM				
ZIF‐8@Au	GOx‐POD	GOx (glucose): V_max_ = 168.4 × 10^−8^ M s^−1^, K_m_ = 1.393 mM	pH = 4.0, *T* = 20 °C	Antibiotic resistance genes: 0309 µM (colorimetric); 0.112 nM (electrochemistry)	Not available.	[92]
		POD (H_2_O_2_): V_max_ = 84.9 × 10^−8^ M s^−1^, K_m_ = 0.322 mM				
α‐Co nanoparticles confined in nitrogen‐doped carbon nanofiber (α‐Co@NCNF)	GOx‐POD	GOx: not available	pH = 4.0, *T* = 25 °C	0.03 µM (glucose); 1.66 µM (H_2_O_2_); and 0.03 µM (GSH).	Not available.	[157]
		POD (TMB): V_max_ = 30.48 × 10^−8^ M s^−1^, K_m_ = 0.173 mM; POD (H_2_O_2_): V_max_ = 106.1 × 10^−8^ M s^−1^, K_m_ = 0.33 mM				
Pyrite nanozyme	OXD‐POD	OXD (GSH): V_max_ = 0.98 × 10^−6^ M s^−1^, K_m_ = 0.66 mM. k_cat_ = 8.34 × 10^4^ s^−1^	pH = 4.5,	10 mg kg^−1^ (intratumor injection), 15 mg/kg (intravenous injection), once every 2 days	Cytotoxicity: negligible cytotoxicity (40 µg mL^−1^). Elimination half‐life (t_1/2_): 50 min.	[158]
		POD (H_2_O_2_): V_max_ = 13.54 × 10^−8^ M s^−1^, K_m_ = 0.041 mM, k_cat_ = 3.67 × 10^4^ s^—1^				
Asymmetric N, S‐coordinated Fe SAzymes (Fe‐S/N‐C)	OXD‐POD	OXD (GSH): V_max_ = 346 × 10^−6^ M s^−1^, K_m_ = 1.0 mM, k_cat_ = 133.59 s^−1^	pH = 7.4, *T* = 42 °C	200 µL, 2 mg mL^−1^ (intravenous injection)	Hemolysis: no significant hemolysis (10‐50 µg mL^−1^)	[17]
		POD (TMB): V_max_ = 1.3 × 10^−6^ M s^−1^, K_m_ = 0.33 mM, k_cat_ = 5.02 s^−1^ POD (H_2_O_2_): V_max_ = 2.4 × 10^−6^ M s^−1^, K_m_ = 8.16 mM, k_cat_ = 9.27 s^−1^	pH = 4.0, *T* = 25 °C			
Single‐atom carbon dots with CuO_4_ coordination environment (Cu‐SLCDs)	SOD‐CAT/POD	SOD: not available	Not available	25 µg mL^−1^ (smear), once every 2 days	Cytotoxicity: negligible cytotoxicity (≤ 100 µg mL^−1^). Hemolysis: not available.	[130]
		CAT (H_2_O_2_): V_max_ = 8.7 mg L^−1^ min^−1^, K_m_ = 1.34 mM	pH = 7.4, *T* = 25 °C			
		POD (TMB): V_max_ = 40.12 × 10^−8^ M s^−1^, K_m_ = 0.18 mM; POD (H_2_O_2_): V_max_ = 69.23 × 10^−8^ M s^−1^, K_m_ = 1.12 mM	pH = 4.5, *T* = 25 °C			
NIR‐responsive inverse oxide/alloy‐structured nanozyme (Co_7_Fe_3_/ZnO@C)	SOD‐CAT/POD	SOD (pyrogallol): V_max_ = 7.14 × 10^−4 ^M s^−1^, K_m_ = 2.07 mM	pH = 8, *T* = 35 °C, NIR irradiation	gel (200 µL, dressing) and 2.5 µL 3% (w/w) H_2_O_2_ mixture	Cytotoxicity: negligible cytotoxicity. Hemolysis: below the international safety threshold of 5%.	[159]
		CAT (H_2_O_2_): V_max_ = 0.55 mg L^−1^ s^−1^, K_m_ = 2.07 mM	pH = 8, *T* = 75 °C, NIR irradiation			
		POD (TMB): V_max_ = 0.85 × 10^−7^ M s^−1^, K_m_ = 5.79 × 10^−2^ mM	pH = 6, *T* = 55 °C, non‐NIR irradiation			
Peptide‐templated manganese dioxide nanozyme (PNzyme/MnO_2_)	SOD‐CAT	SOD: Specific activity (SA) = 2139.86 U mg^−1^	pH = 7.4, *T* = 37 °C	10 µg mL^−1^ (tail vein injection), (IC_50_ = 59.04 µg mL^−1^)	Cytotoxicity: negligible cytotoxicity (25 mg mL^−1^). Hemolysis: no significant hemolysis (40‐200 µg mL^−1^). Elimination half‐life (t_1/2_): 4.63 ± 0.50 h.	[160]
		CAT (H_2_O_2_): V_max_ = 5.22 × 10^−2^ M s^−1^, K_m_ = 4.9 mM, SA = 255.77 U mg^−1^				
Pt@CNDs	SOD‐CAT	SOD: SA = 12 605 U mg^−1^	pH = 7.4	1.5, 2.5, 4 mg kg^−1^ (liver injury, intravenous injection); 2.5 and 5 µg kg^−1^ (ear inflammation, subcutaneous injection)	Cytotoxicity: < 5% (100 µg mL^−1^). Hemolysis: < 3.2% (10‐1000 µg mL^−1^). Elimination half‐life (t_1/2_): 48 min.	[161]
		CAT (H_2_O_2_): V_max_ = 24.82 × 10^−10^ M min^−1^, K_m_ = 42.84 mM, SA = 3172 U mg^−1^				
Hexaiminohexaazatrinaphthalene COF with ruthenium coordination (S‐HACOF‐Ru)	SOD‐CAT	SOD: not available	not available	20 µL, 1 mg/mL (periodontitis, intragingival injection); 15 µL, 1 mg/mL (rheumatoid arthritis, intraarticular injection), twice a week	Cytotoxicity: negligible cytotoxicity (≤ 5 µg mL^−1^). Hemolysis: not available.	[162]
		CAT (H_2_O_2_): V_max_ = 43.9 × 10^−6^ M s^−1^, K_m_ = 273.2 mM, turnover number (TON) = 4.25 s^−1^	pH = 7.4			
Ru‐hydroxide	SOD‐CAT	SOD: not available	not available	100 µg mL^−1^ (smear)	Cytotoxicity: negligible cytotoxicity. Hemolysis: not available.	[163]
		CAT (H_2_O_2_): V_max_ = 63.29 × 10^−6^ M s^−1^, K_m_ = 69.59 mM, TON = 12.06 s^−1^	pH = 7.4			
NiFeMnCu‐LDH	CAT‐OXD	CAT (H_2_O_2_): V_max_ = 23.2 × 10^−8^ M s^−1^, K_m_ = 78.8 mM	pH = 7.5	5 mg kg^−1^, Once every 3 days (tail vein injection)	Cytotoxicity: negligible cytotoxicity (300 µg mL^−1^). Hemolysis: 300 µg/mL (< 1.9%).	[164]
		OXD (TMB): V_max_ = 1.09 × 10^−8^ M s^−1^, K_m_ = 0.02 mM,	pH = 3.5			
Sulfur‐modified bimetallic single‐atom nanozyme (S‐FeCo‐NC)	CAT‐OXD	CAT: not available	not available	Tetracycline: 0.22 µM (colorimetric); 1.68 nM (electrochemistry)	Not available	[55]
		OXD (2,4‐DP): V_max_ = 3.1 × 10^−8^ M s^−1^, K_m_ = 0.15 mM, SA = 104.4 U g^−1^	pH = 7.4, *T* = 25 °C,			
PdCu‐pNS capsules	CAT‐OXD	CAT (H_2_O_2_): V_max_ = 40.36 × 10^−8^ M s^−1^, K_m_ = 37.55 mM,	pH = 5, under 1064 nm laser irradiation	200 µL, 25 mg/kg (intravenous injection) (IC_50_ = 150 µg mL^−1^, approximately)	Cytotoxicity: < 5% (0–180 µg mL^−1^). Hemolysis: < 3.2% (10–1000 µg mL^−1^). Bood circulation half‐life (t1/2β): 4.32 h	[149]
		OXD: not available	pH = 4.5–5.5			
Two‐dimensional (2D) Pd@Ir nanosheets	OXD‐CAT	OXD (uric acid): V_max_ = 3.7 × 10^−7^ M s^−1^, K_m_ = 3.0 × 10^−2^ mM	Not available	25 µL, 100 µg mL^−1^ (knee‐joint injection)	Cytotoxicity: < 30% (500 µg mL^−1^). Hemolysis: no significant hemolysis (15‐200 µg mL^−1^)	[96]
		CAT (H_2_O_2_): V_max_ = 9.6 × 10^−7^ M s^−1^, K_m_ = 36 mM				

### 5.1 GOx‐POD

The cascade reaction consisting of GOx and POD is one of the most common nanozymatic reaction systems. In this cascade catalytic system, H_2_O_2_ produced by the glucose oxidation reaction serves as the substrate for POD. Leveraging the high stability and cost‐effectiveness of nanozymes, numerous all‐nanozyme GOx‐POD systems have been developed. In the reported studies, these GOx‐POD cascade systems are primarily constructed using nanozymes with dual enzymatic activities. For example, some nanozymes exhibiting both GOx‐like and POD‐like activities have been synthesized by loading gold nanoparticles, which possess GOx‐like activity, onto materials with POD activity.^[^ ^92^, ^165^, ^166^ ^]^ For example, Kong et al. developed AuNPs@ZIF‐8‐C nanozyme with GOx‐POD cascade function by anchoring AuNPs onto carbonated zeolitic imidazolate framework‐8 (ZIF‐8‐C), which were used for glucose monitoring in sweat.^[^ ^167^ ^]^ In the AuNPs@ZIF‐8‐C, the AuNPs acted as GOx mimicked and ZIF‐8‐C served as a POD mimic. Similarly, Deng et al. synthesized a multi‐nanozyme system (MSN‐Au) with both GOx‐like and POD‐like catalytic activities by integrating Au, nanoparticles (Au NPs) onto mesoporous silica nanoparticles (MSN).^[^ ^155^ ^]^ The Au NPs catalyzed the conversion of glucose into H_2_O_2_ and gluconic acid via GOx‐like activity and subsequently transformed H_2_O_2_ into •OH via POD‐like activity, which effectively destroyed bacteria. Additionally, when MSN‐Au was loaded with MnCO, it produced carbon monoxide (CO) in response to H_2_O_2_, and the combined action of CO and Au NPs exerted a synergistic anti‐inflammatory effect in macrophages challenged by lipopolysaccharides.

Furthermore, various inorganic nanomaterials such as noble metals,^[^ ^85^, ^105^, ^156^ ^]^ MOF,^[^ ^157^ ^]^ modified carbon nitride,^[^ ^168^ ^]^ transition metal phosphides (TMPs),^[^ ^169^ ^]^ metallic oxides,^[^ ^170^, ^171^, ^172^ ^]^ also exhibit dual enzymatic activities similar to GOx and POD. For instance, Geng et al. developed a liposomal nanoplatform (Ir/CeO_2_@Lipo) by anchoring iridium (Ir) single atoms onto CeO_2_ quantum dot (QDs) nanoislands, forming Ir/CeO_2_ single‐atom nanoislands (SANIs) with strong metal–support interactions.^[^ ^154^ ^]^ The abundant oxygen vacancies in CeO_2_ and the formation of Ir–O–Ce interfacial sites facilitated efficient electron transfer, thereby enhancing GOx‐ and POD‐like self‐cascade catalytic activity. Compared to conventional nanozymes, Ir/CeO_2_@Lipo exhibited superior ROS generation and demonstrated potent tumor ablation effects in vivo, offering a promising strategy for catalytic tumor therapy. In another work, Yang et al. synthesized MOF‐derived nanozyme (α‐Co@NCNF) with a dual enzyme‐like activity by confined α‐cobalt nanoparticles into the nitrogen‐doped carbon nanofiber.^[^ ^157^ ^]^ The α‐Co@NCNF nanozyme exhibited GOx‐ and POD‐like activity and was utilized to construct colorimetric biosensing for monitoring glucose, H_2_O_2_, and GSH.

### 5.2 OXD‐POD

In addition to classical GOx‐POD cascade systems, various cascade systems combining other OXD‐like nanozyme with POD‐like nanozyme, have been successfully constructed, including flavin oxidase (FOx)‐POD,^[^ ^173^ ^]^ NOX‐POD,^[^ ^174^, ^175^, ^176^ ^]^ uricase (UOx)‐POD,^[^ ^25^, ^177^ ^]^ GSHOx‐POD,^[^ ^17^, ^158^, ^178^, ^179^ ^]^ polyphenol oxidase (PPO)‐POD.^[^ ^180^ ^]^ For example, by in situ reduction of Pt clusters on Fe@C under UV irradiation, Zheng et al. synthesized iron‐doped carbon (Fe@C) nanozyme, which demonstrated dual NOX and POD‐like characteristics.^[^ ^174^ ^]^ In their work, NOX‐mimic Pt/Fe@C oxidizes NADH to NAD^+^ while reducing O_2_ to H_2_O_2_, whose yield correlates with NADH concentration. The resulting H_2_O_2_ then oxidates 3,3′,5,5′‐tetramethylbenzidine (TMB) via Pt/Fe@C's POD‐like activity to generate detectable signals. Compared to the traditional method based on the combination of natural NOX and horse radish peroxidase (HRP), the developed cascade nanozyme can catalyze multistage reactions in a singular setup, simplifying detection processes and enhancing sensitivity. In another work, Zhu et al. synthesized a carbon nitride nanozyme featuring atomic K‐coupled cyano sites (K─CN) with both FOx‐like and POD‐like activities by a template‐confined polymerization strategy.^[^ ^173^ ^]^ Each activity of K─CN not only performed its functions for specific pollutants but also combined to improve efficiency. Based on the K─CN nanozyme, a nanozyme metabolism system was reported to realize broad‐spectrum protection from air pollution.

### 5.3 GOx‐CAT

The combination of GOx‐mimicking nanozymes and CAT‐mimicking nanozymes is mainly used to consumed glucose to generate lots of H_2_O_2_ and gluconic acid; the produced H_2_O_2_ is then decomposed into water and oxygen by CAT‐mimicking nanozymes. This cascade can not only achieve supplemental O_2_ supply and down‐regulated glycolysis to alleviate hypoxia and high glucose metabolism, but also downregulate pH and reduce ROS to achieve microenvironmental regulation.^[^ ^181^, ^182^ ^]^ For example, Shuai et al. reported a bifunctional nanosystem (MnZ@Au) using Mn^2+^‐doped CDs as the core and ZIF‐8 as the shell, with ultrasmall AuNPs anchored on the surface.^[^ ^183^ ^]^ This system catalyzed glucose consumption, generating H_2_O_2_ and gluconic acid, while releasing Mn‐CDs to drive CAT‐like reactions, producing O_2_, which enhanced photodynamic therapy (PDT) efficacy by alleviating tumor hypoxia and reducing glucose metabolism. Similarly, a novel nanosystem (Cyano@Au@Ir) was developed using cyanobacteria as carriers loaded with Au NPs (GOx‐like activity) and Ir NPs (CAT‐like activity) to address diabetic retinopathy.^[^ ^184^ ^]^ The Au NPs nanozyme first degrades glucose into hydrogen peroxide, which is further decomposed into H_2_O and O_2_ by the Ir NPs to complete the cascade hypoglycemic reaction.

### 5.4 CAT‐OXD

In this cascade type, nanozymes with CAT‐like catalytic activity convert H_2_O_2_ to anode O_2_. Subsequently, generated O_2_ is subsequently utilized to promote the oxidation of substrates by nanozymes simulating the activity of the OXD activity. This system achieves a highly effective antitumor effect by increasing ROS accumulation and regulating oxidative stress.^[^ ^185^, ^186^, ^187^, ^188^ ^]^ For instance, a bifunctional nanozyme with Co−N coordination on N‐doped porous carbon (Co‐SAs@NC) was developed for synergistic tumor therapy with doxorubicin (DOX).^[^ ^189^ ^]^ Acting as a nanozyme both with CAT‐like and OXD‐like activities, they efficiently catalyze the conversion of H_2_O_2_ and O_2_ into a large amount of cytotoxic O_2_^•−^ radicals. Na et al. developed an aptamer‐functionalized Pd@MoO*_3_–x* nano‐hydrangea (A‐Pd@MoO*_3_–x* NH) for high‐efficiency cancer therapy.^[^ ^190^ ^]^ This innovative nanozyme can simultaneously expose dual active centers (the Pd(111) and Pd(100) surface facets) of the CAT‐like (decomposing H_2_O_2_ into O_2_) and OXD‐like (converting O_2_ into O_2_^•−^) nanozyme for catalyzing cascade reactions.

### 5.5 PEH‐OXD

Phosphoester hydrolase (PEH)‐OXD cascade catalytic nanozymes have recently attracted significant attention, as they provide a transformative approach for designing next‐generation organophosphorus pesticide biosensors based on catalytic conversion. In these cascade systems, the hydrolytic reaction is catalyzed by hydrolase‐like nanozymes to generate specific intermediate products, which subsequently act as substrates for OXD‐like nanozymes to produce detectable signals. For example, Ye et al. synthesized a Cu/Ce‐MOF‐808 nanozyme as a cascade catalytic element with hydrolase‐like and catechol oxidase‐like activities.^[^ ^191^ ^]^ With isocarbophos as the representative, the organophosphorus pesticide (OP) was first hydrolyzed by Cu/Ce‐MOF‐808 to generate isopropyl salicylate, which was further oxidized via catechol oxidase‐like activity of Cu/Ce‐MOF‐808 to form a colored quinoneimine complex with 4‐aminoantipyrine (4‐AAP) under light irradiation. Similarly, Ce‐MOF‐based nanozymes (HMUiO‐66(Ce)) with PEH‐ and OXD‐like dual activities have been engineered to achieve self‐cascade recognition of profenofos through hydrolysis and selective oxidative coupling, enabling sensitive and selective colorimetric detection.^[^ ^192^ ^]^ Collectively, these studies demonstrated the versatility and efficiency of hydrolase–oxidase cascade nanozymes for substrate recognition and signal amplification, offering promising strategies for the development of integrated biosensing platforms.

### 5.6 SOD‐CAT

Overproduced ROS production induces oxidative stress, leading to numerous acute and chronic inflammatory diseases, such as cardiovascular, neural, kidney, and immune diseases, as well as cancers. Using the cascade effects of SOD and CAT to mitigate elevated oxidative stress and ROS levels is an effective strategy to deal with these diseases. In a typical SOD‐CAT cascade reaction, SOD converts the highly reactive O_2_^•−^ into H_2_O_2_; subsequently, CAT decomposes the H_2_O_2_ into nontoxic oxygen and water. Recently, many catalytic nanomaterials with SOD‐like and CAT‐like activities have been investigated, such as Ce‐based,^[^ ^193^, ^194^, ^195^, ^196^, ^197^, ^198^ ^]^ Cu‐based,^[^ ^199^, ^200^, ^201^ ^]^ and Mn‐based nanozymes.^[^ ^160^, ^202^, ^203^, ^204^, ^205^, ^206^, ^207^ ^]^ These nanomaterials have shown great promise in the treatment of ROS‐related diseases. For example, Jiang et al. synthesized a Cu‐LDH nanozyme with dual enzyme‐like (SOD‐CAT) activities and integrated it with NO‐releasing molecules to create the advanced nanoreactor Cu‐LDH@GSHNO.^[^ ^199^ ^]^ This system efficiently converted O_2_^•−^ to H_2_O_2_ and then to O_2_, while also releasing GSH and NO to scavenge ROS and normalize vascular function, demonstrating strong potential in treating retinal neovascular diseases. As a typical antioxidant nanomaterial, cerium‐based nanozyme, as a scavenger of ROS has attracted particular attention by mimicking SOD/CAT activities, owing to its reversible valence state conversion between Ce^3+^ and Ce^4+^. For example, Zhang et al. synthesized dextran‐coated cerium oxide (D‐CeO_2_) nanozyme with SOD and CAT activities.^[^ ^193^ ^]^ D‐CeO_2_ effectively scavenged ROS and reduced the levels of pro‐inflammatory cytokines (IL‐1β, IL‐6, TNF‐α, and iNOS), thereby protecting cells from oxidative damage induced by H_2_O_2_. However, CeO_2_ nanospheres have limited enzymatic activity that hinders further application in catalytic therapy. Zhong et al. synthesized PtCuOX/CeO_2_‐X nanozymes by introducing bimetallic Cu and Pt into CeO_2_ nanospheres, enhancing oxygen vacancies and achieving uniform metal dispersion.^[^ ^208^ ^]^ This defect‐engineering approach improved SOD/CAT‐like activities through enhanced electron transfer, increased intermediate adsorption, and reduced reaction activation energy. Recently, to address the issues of poor biosafety, limited targeting, and low retention of nanozymes, Zheng et al. developed a degradable cerium–tannic acid (CeTA) nanozyme integrated with a self‐assembling peptide (CeTA‐K1tkP), forming a nasal inhalable platform for treating viral pneumonia.^[^ ^209^ ^]^

Noble metals have also been designed to defend oxidative damage via their intrinsic CAT‐, SOD‐like activities.^[^ ^63^, ^161^, ^210^ ^]^ For instance, Liu et al. reported a novel Pt@CNDs nanocomposite with SOD‐ and CAT‐like activities by integrating carbon nanodots (CNDs) with platinum nanoparticles (PtNPs),^[^ ^161^ ^]^ which could be utilized as an excellent antioxidant nanozyme for protecting biosystems from ROS‐induced damages. The Pt@CNDs exhibited much higher catalytic activities compared with individual PtNPs or CNDs due to the synergistic effects between PtNPs and CNDs. Cheng et al. synthesized covalent organic framework (COF)‐based artificial metalloantioxidases (S‐HACOF‐Ru) with excellent SOD/CAT‐like catalytic activities and broad spectrum ROS scavenging capabilities.^[^ ^162^ ^]^ Similarly, Shi et al. synthesized bimetallic ruthenium–cobalt (RuCo) nanosheets that effectively simulate the SOD‐CAT cascade.^[^ ^113^ ^]^ Notably, after 4 h of exposure to simulated gastric fluid (SGF), RuCo nanosheets maintained stable SOD‐CAT‐like cascade catalytic activity. This research presents a gastric‐acid‐stabilized antioxidative nanocatalytic platform for the efficient treatment of inflammatory diseases of the digestive system.

### 5.7 All in One

It is worth pointing out that due to the multiple properties of disease microenvironments, nanozymes with more enzyme mimetic activities have been designed to remodel the disease microenvironment and achieve multimodal synergistic therapy. Based on the number of catalytic functions, these nanozymes can be classified as three‐in‐one, four‐in‐one, five‐in‐one, or six‐in‐one systems (**Table** **2**). Given that the majority of currently reported nanozymes predominantly mimic redox enzymes, their therapeutic functions in practice are largely governed by two primary mechanisms: prooxidative and antioxidative pathways. Among them, prooxidative therapy mainly uses multi‐enzyme cascades to increase ROS production, accompanied by glucose and GSH consumption. For example, Zhou et al. developed a cascade‐augmented nanoimmunomodulator (CMZM) with four enzyme‐like activities (SOD, CAT, POD and GSHOx) to inhibit tumor growth by generating H_2_O_2_, O_2_, and •OH and consuming GSH.^[^ ^211^ ^]^ In contrast, antioxidant therapy involves the use of antioxidant enzyme activity to remove ROS and regulate the oxidative stress state in cells. For example, Wei et al. constructed a self‐cascading antioxidant nanozyme LiMn_2_O_4_, which not only exhibited SOD‐like activity, but also CAT‐like and GPx‐like activities. The nanozyme demonstrated the outstanding antioxidant capacity at the cellular level and in the treatment of IBD in mice.^[^ ^120^ ^]^ Similarly, Yang et al. reported an iodine–copper–zinc covalent‐doped carbon dot (Cu,Zn,I‐CD) nanozyme with high overall SOD‐like, CAT‐like, and GPx‐like triple antioxidant nanozyme activities.^[^ ^212^ ^]^ Owing to the triple enzyme‐like activities, Cu,Zn,I‐CDs were used to treat ulcerative colitis by scavenging overproduced ROS.

**Table 2:** A summary of multienzyme‐like nanozymes‐based biomimetic cascade catalytic systems.

Number of mimicking enzyme activities	Cascade nanozymes	Kinds of mimicking enzyme activities	Treatment mechanism	Refs.
Three‐in‐one	Co_7_Fe_3_/ZnO@C	POD‐CAT‐SOD	ROS generation	[159]
	Co NSs	POD‐CAT‐SOD	ROS scavenging	[213]
	PBzymes	POD‐CAT‐SOD	ROS scavenging	[214]
	Mn@Bi_2_Se_3_@RGE‐Exos	POD‐OXD‐CAT	ROS generation	[215]
	AuMnCu	POD‐OXD‐CAT	ROS generation GSH consumption	[216]
	H‐MoN_5_@PtN_4_/C	POD‐OXD‐CAT	ROS generation GSH consumption	[217]
	AuSAN	POD‐OXD‐CAT	ROS generation	[218]
	CeO_2_Mn_1.08_O_x_	POD‐OXD‐CAT	ROS generation GSH consumption	[172]
	CuSACO	POD‐OXD‐CAT	ROS generation	[219]
	MoO_x_‐Rh	POD‐OXD‐CAT	ROS generation	[220]
	Au–Pt NPs	POD‐GOx‐CAT	ROS generation GSH consumption	[26]
	gCM@MnAu	POD‐GOx‐CAT	ROS generation	[15]
	HABT‐C	POD‐GOx‐CAT	ROS generation	[16]
	Ti_3_C_2_T_x_‐Au‐PEG	POD‐GOx‐CAT	ROS generation glucose consumption	[165]
	Cu, Zn, I‐CDs	CAT‐SOD‐GPx	ROS scavenging	[212]
	CDs	CAT‐SOD‐GPx	ROS scavenging	[221]
	GSH‐CDs	CAT‐SOD‐GPx	ROS scavenging	[222]
	ZnMn_2_O_4_	CAT‐SOD‐GPx	ROS scavenging	[120]
	Ru‐hydroxide	CAT‐SOD‐GPx	ROS scavenging	[163]
	PCN@ZF‐HA (PZFH)	OXD‐SOD‐POD	ROS generation	[223]
	MnO_x_/CeBTC	OXD‐SOD‐POD	ROS generation	[224]
	FeCu‐DA	POD‐CAT‐GSHOx	ROS generation GSH consumption	[225]
	IB@Fe‐ZIF8@PDFA	POD‐GSHOx‐NOX	ROS generation GSH consumption	[226]
	Cu_2_O@Au	POD‐GOx‐GPx	ROS generation GSH consumption	[227]
	Mn–N–C	UOx‐SOD‐ CAT	UA degradation ROS scavenging	[228]
Four‐in‐one	CMZM	SOD‐POD‐CAT‐ GSHOx	ROS generation GSH consumption	[211]
	Mn_3_O_4_@g‐C_3_N_4_	POD‐OXD‐CAT‐OXD	ROS scavenging	[229]
	RuX nanozymes	POD‐OXD‐CAT‐GSHOx	ROS generation GSH consumption	[110]
	2D NiCoO_x_	POD‐OXD‐CAT‐GSHOx	ROS generation GSH consumption	[230]
	MoCu DAzyme	POD‐OXD‐GSHOx‐NOX	ROS generation GSH consumption	[231]
	Fe_2_NC@Selenium	SOD‐CAT‐OXD‐GPx	ROS scavenging	[53]
	PdCu‐pNS	POD‐CAT‐OXD‐GPx	ROS generation GSH consumption	[149]
	NiFeMnCu‐LDH	POD‐CAT‐OXD‐GPx	ROS generation GSH consumption	[164]
	Au–MoS_2_	POD‐CAT‐SOD‐GOx	ROS generation glucose consumption	[232]
	RuO_2_‐PVP NPs	POD‐CAT‐SOD‐APx‐TPx^a)^	ROS scavenging	[233]
Five‐in‐one	Ca_2_Mn_8_O_16_	POD‐CAT‐OXD‐GPx‐GOx	ROS generation GSH consumption	[24]
	Au/Cu_1.6_O/P–C_3_N_5_/Arg@HA	SOD‐CAT‐GOx‐POD‐NOS^b)^	ROS scavenging glucose consumption	[21]
	RuO_2_	POD‐CAT‐SOD‐GPx‐OXD	ROS scavenging	[234]
	RuO_2_‐PVP NPs	POD‐CAT‐SOD‐APx‐TPxa)	ROS scavenging	[233]
Six‐in‐one	CaHCF NPs	POD‐CAT‐SOD‐GPx‐TPx‐APx	ROS scavenging	[235]
	ReSe_2_ nanoflowers	POD‐CAT‐SOD‐SOD‐GPx‐NOX	ROS generation GSH consumption	[236]

^a)^ APx: ascorbate peroxidase, TPx: thiol peroxidase;

^b)^ NOS: nitric oxide synthase.

Importantly, the therapeutic applicability of multi‐active nanozymes cannot be simply inferred from the number or type of enzyme‐like activities they possess. Some studies have demonstrated that even for cascade nanozymes with the same set of enzyme‐like activity, they may exhibit markedly different performances in practical applications. For instance, Wei et al. developed a near‐infrared (NIR)‐activated inverse oxide/alloy‐structured nanozyme (Co_7_Fe_3_/ZnO@C). This nanozyme exhibits three distinct catalytic activities of POD, CAT, and SOD.^[^ ^159^ ^]^ Specifically, the nanozyme exhibits POD‐like activity in acidic wound conditions to generate bactericidal •OH, while NIR irradiation triggers SOD‐like, CAT‐like, and hydroxyl radical antioxidant capacity (HORAC) activities to eliminate residual ROS and convert H_2_O_2_ into O_2_, alleviating hypoxia and promoting angiogenesis. This cascaded network dynamically balances ROS production (POD) and scavenging (NIR‐driven SOD/CAT/HORAC), eradicating bacteria while resolving inflammation. In contrast, Zhang et al. synthesized 2D cobalt hydroxide oxide nanosheets (Co NSs) that also exhibited POD‐like, CAT‐like and SOD‐like activities, but primarily served as an antioxidant system.^[^ ^213^ ^]^ DFT calculations pinpointed that CAT played a leading role among three catalytic activities of Co NSs under physiological conditions, followed by SOD‐like and POD‐like activity. These findings demonstrated that even nanozymes with the same types of enzyme‐mimicking activities may exhibit different therapeutic functions. This difference ultimately depends on the dominant catalytic activity under specific micro‐environmental conditions. Therefore, accurate evaluation of nanozyme function requires in‐depth mechanistic understanding rather than relying solely on the nominal count of enzymatic functions.

### 5.8 Refined Classification of Cascade Nanozyme Systems

Based on the above classification formed by the simple superposition of enzyme activity functions, introducing additional classification dimensions would allow a more nuanced interpretation of how cascade nanozyme systems are constructed and function. Therefore, to further understand the reaction essence and design logic of cascade nanozyme systems, the following sections analyze cascade reactions from several perspectives that together inform the development of controllable, biomimetic catalytic platforms.

#### 5.8.1 Classification Based on Reaction Intermediates

Given that oxidoreductases are currently the main category in current nanozyme studies, a useful initial partition is based on the identity of key intermediates. Most cascade nanozyme systems rely on H_2_O_2_ as the key intermediate bridging two enzymatic steps. In typical examples such as GOx‐POD, OXD‐POD, GOx‐CAT, and SOD‐CAT, the first reaction produces H_2_O_2_, which is immediately consumed by a downstream POD or CAT. These H_2_O_2_‐mediated cascades are widely applied in fields such as biosensing and tumor treatment due to their clear reaction pathways, easily assayed products, and strong signal‐amplification characteristics. In contrast, a few cascade systems utilize O_2_ as the intermediate. For instance, in CAT‐OXD cascade systems, CAT decomposes H_2_O_2_ to generate O_2_, which is then consumed by OXD. These O_2_‐mediated cascade reactions can locally regulate O_2_ levels, thereby maintaining continuous catalytic activity under hypoxic conditions and providing an important strategy for the construction of self‐oxygen‐supplying catalytic systems.

#### 5.8.2 Classification Based on Reaction Directionality and Coupling Relationships

Beyond the intermediate characteristics, nanozyme cascade systems can also be classified according to the directionality and coupling relationship among the individual reactions, into unidirectional cascade reaction and feedback cascade reaction. In unidirectional cascade reactions (e.g., GOx‐POD, OXD‐POD, SOD‐CAT, PEH‐OXD), the product of the first enzymatic step is the substrate of the second step, but the final product cannot be reused in the upstream step, thus forming an irreversible cascade catalysis. Taking the GOx‐POD system as an example, H_2_O_2_ was produced by the glucose oxidation reaction, and then POD decomposes the H_2_O_2_ into •OH. Because the generated •OH cannot be utilized by GOx, the system exhibits irreversible and unidirectional reaction kinetics. This linear cascade enables continuous ROS generation and signal enhancement, making it suitable for biosensing and chemodynamic therapy that require enhanced oxidation.^[^ ^167^, ^170^, ^172^ ^]^ In feedback cascade systems, downstream products can be used to replenish the substrate of the initial reaction, thereby forming a self‐regulating catalytic cycle. For example, in the GOx‐CAT system, GOx oxidizes glucose with O_2_ to generate H_2_O_2_, and then CAT subsequently decomposes H_2_O_2_ into O_2_.^[^ ^181^, ^182^ ^]^ Under certain conditions, the regenerated O_2_ can be reutilized by GOx. The level of O_2_ and H_2_O_2_ is balanced by the cascade reaction, which is conducive to the adaptive regulation of the dynamic microenvironment. The feedback characteristic of this type of cascading system simulates the steady‐state control mechanism of biological enzyme systems, and is crucial for designing responsive and self‐adjusting biomimetic catalytic systems. In summary, unidirectional cascades amplify signals through irreversible reaction chains, whereas feedback cascades achieve dynamic responsiveness and environmental adaptability via closed‐loop regulation, jointly broadening the functional diversity and complexity of artificial catalytic networks.

#### 5.8.3 Classification Based on ROS Regulation Behavior

The capacity of cascade nanozyme systems to regulate ROS is central to their application potential. From the perspective of ROS evolution, these cascade systems can further be categorized into ROS‐generating, ROS‐scavenging, and ROS‐interconversion types. In ROS‐generating cascades (e.g., GOx‐POD, OXD‐POD), the reaction sequence ultimately yields highly reactive ROS, thereby increasing oxidative stress for antibacterial or anticancer purposes.^[^ ^154^, ^155^ ^]^ ROS‐scavenging cascade systems operate in the opposite manner. A representative example is SOD‐CAT cascade reaction, which sequentially catalyzes O_2_^•−^ and ultimately converts it into non‐toxic or inert products, thus protecting biological systems from oxidative injury.^[^ ^161^, ^209^ ^]^ The third type is the ROS‐interconversion cascades, which focus on mutual transformation among various redox species to finely tune the reactivity and cytotoxicity of reactions. For example, in SOD‐POD cascades, O_2_^•−^ is first converted into H_2_O_2_ and then into the more reactive •OH;^[^ ^237^, ^238^ ^]^ similarly, CAT‐OXD cascade systems can transform H_2_O_2_ into O_2_‐derived radicals with higher reactivity,^[^ ^186^, ^188^, ^189^ ^]^ enabling controllable adjustment of the oxidative microenvironment. Overall, these three types of cascades offer complementary functions in ROS production, elimination, and transformation. By precisely engineering reaction pathways and coupling modes, cascade nanozyme systems can realize integrated catalytic strategies ranging from enhanced oxidative therapy to the maintenance of physiological redox homeostasis.

## 6 Applications

### 6.1 Biosensing

Cascade systems offer superior signal amplification and selectivity by continuously converting substrates into signaling molecules and introducing sequential “logical” recognition steps. Compared with the natural enzyme‐involved cascade system, the all‐nanozyme cascade system exhibits many advantages, including superior cascade catalytic efficiency and high stability. In view of these advantages, nanozyme‐constructed cascade systems possess highly effective cascade catalytic performance for the transformation of substrates and remarkably enhance the signal amplification intensity, resulting in their widespread applicability for biosensing.

#### 6.1.1 Selectivity Enhancement by Cascade Nanozymes

**Figure 5:**
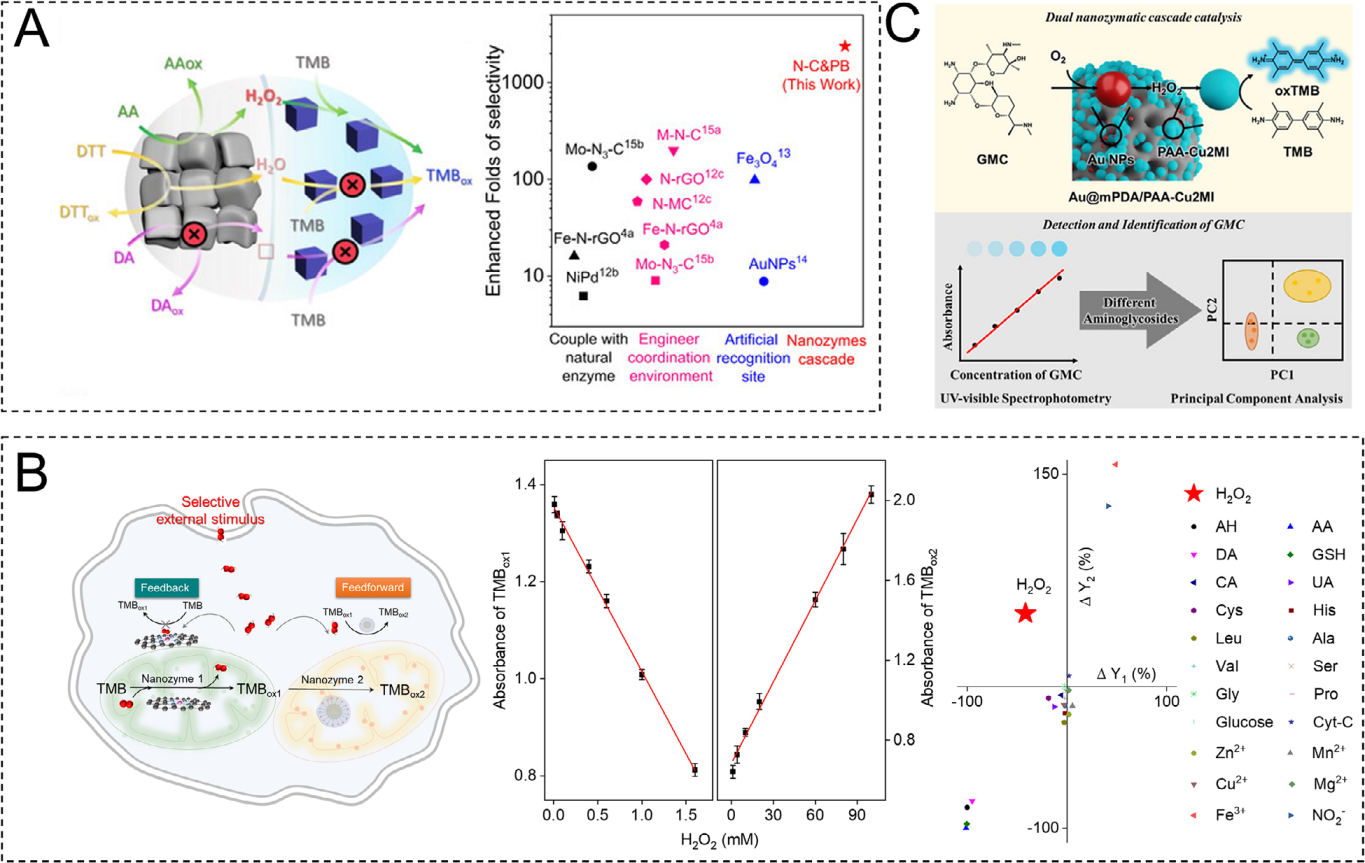
A) Schematic of nanozymes cascade system consisting of oxidase‐like (dark cages) and peroxidase‐like (blue cubes) nanozyme. Comparison of the enhanced selectivity by nanozymes reported herein with those described in the literature. Reproduced with permission.^[^ ^22^ ^]^ Copyright 2022, Wiley‐VCH. B) Schematic of cascade nanozymatic network within the microfluidic chip as a simplified artificial cell. Linear and selective response to H_2_O_2_. Reproduced with permission.^[^ ^70^ ^]^ Copyright 2023, Royal Society of Chemistry. C) Schematic of detection and identification of multiple AGs by cascade reaction. Reproduced with permission.^[^ ^243^ ^]^ Copyright 2025, American Chemical Society.

Selectivity is a critical trait for biosensors, which discriminates the target from interferents. By requiring an analyte to participate in two coupled reactions, cascade nanozymes can greatly improve selectivity versus one‐step systems. In effect, the cascade acts as a two‐step filter, strongly favoring the targeted analyte. More generally, cascades can magnify the differences between analytes and interferences. In the typical case, only the intended substrate is fully converted to product through both steps, whereas interferents are largely blocked after the first or second step.^[^ ^239^, ^240^, ^241^, ^242^ ^]^ A representative study was reported by Zhang et al. wherein an oxidase‐like nanozyme and a peroxidase‐like nanozyme were cascaded to improve the reaction selectivity in transforming the substrate into the targeted product by more than 2000 times (**Figure** **5**A).^[^ ^22^ ^]^ Subsequently, these workers introduced the concept of “cascade nanozymatic network” to enhance substrate selectivity through diverse responses to a single stimulus (Figure 5B).^[^ ^70^ ^]^ As a proof‐of‐concept, the nanozymatic network was confined in a microfluidic chip as a simplified artificial cell. Owing to the unique responses of variable intensities and modes, the proposed cell mimics exhibited highly selectivity and linearity in perception of H_2_O_2_ stimulus against more than 20 interferences. Recently, Chen et al. proposed a compartmentalized dual‐nanozyme cascade composite (Au@mPDA/PAA‐Cu2MI, AmPC) for the colorimetric detection of aminoglycoside antibiotics (AGs).^[^ ^243^ ^]^ The AmPC composite exhibited both analogue GOx‐like and POD‐like catalytic activities. In this cascade system, AGs with sugar moieties can serve as the initial substrate, while the generated H_2_O_2_ functions as a secondary substrate for subsequent colorimetric detection (Figure 5C). Non‐AG interferents (lacking the sugar structure) cannot trigger the first step and thus produce little signal. This strategy enables the nanozyme‐based system to selectively recognize target analytes via a cascade reaction mechanism. Furthermore, by employing principal component analysis (PCA) to distinguish among different AGs, the platform allows for simultaneous detection and classification of antibiotics, demonstrating strong potential for on‐site analytical applications. Su et al. proposed a novel nanozyme (Cu@Zr) with all‐in‐one dual enzyme and fluorescence properties by simple self‐assembly.^[^ ^23^ ^]^ Based on its dual enzymatic activities of phosphatase and laccase, a nanozyme cascade sensor with disodium phenyl phosphate (PPDS) as substrate was developed. Specifically, phosphatase cleaves the P─O bond of PPDS to produce colorless phenol, which is then oxidized by laccase and complexed with the chromogenic agent 4‐aminoantipyrine (4‐AP) to produce red quinoneimine. Strikingly, the product NH_3_ of urease and urea could interact with Cu@Zr, accelerating the electron transfer rate and thus enhance the catalytic activity of Cu@Zr, which correspondingly causes the fluorescence of Cu@Zr to be quenched by the generated quinoneimine. Thus, the colorimetric and fluorescence dual‐mode strategy for sensitive urease analysis with limit of detections (LODs) of 3.56 and 1.83 U L^−1^ was established by the proposed cascade sensor.

#### 6.1.2 Signal Amplification by Cascade Nanozymes

Cascade nanozymes can amplify and enhance signals by sequentially generating or converting intermediate products through multistep reactions, thereby offering unique advantages in improving sensor performance. For example, Wang et al. have boosted detection sensitivity of GSH by engineering a three‐enzyme cascade reaction system.^[^ ^244^ ^]^ In this work, CoFe Prussian blue analogue (CoFePBA) demonstrated excellent multi‐enzyme cascade catalytic activity that supported an O_2_→O_2_^•−^→H_2_O_2_→•OH cascade reaction. The multiple steps produced hydroxyl radicals that oxidized a colorimetric dye, enhancing signal output. Utilizing the inhibitory effect of GSH on multi‐enzyme cascade catalytic activity, a highly efficient and rapid colorimetric sensor was designed for the sensitive detection of GSH, with a detection range of 0.5–160 µM and a limit of detection of 0.15 µM. Recently, a multifunctional nanozyme, denoted Pt NPs/CoSAs@NC, was engineered to motivate the in situ cascade catalytic polymerization of dopamine (DA) for constructing a highly sensitive photocurrent‐polarity‐switching PEC biosensing platform.^[^ ^245^ ^]^ Mechanistically, O_2_ and reactive oxygen species (O_2_^•−^ and •OH) generated by the cascade reactions converted dopamine into a conductive polydopamine, which served as the signal reporter for signal amplification and photocurrent polarity switching. As reported, this nanozyme‐based cascade catalytic polymerization strategy yielded sensitively quantifying protein tyrosine phosphatase 1B (PTP1B) with an ultralow limit of detection (0.04 fM), wide linear range (0.1 fM–0.1 µM).

Although cascade nanozyme systems demonstrate significant advantages in signal amplification, they must nevertheless balance substrate effects, stability, and amplification performance when operating in complex biological environments. This is because biological matrices usually contain a wide variety of proteins, lipids, and biomolecules that may compete with or scavenge the reactive intermediates generated during the cascade process. These factors often disrupt the analytical signal and elevate background noise, ultimately reducing detection sensitivity and stability of sensors. Therefore, some sensors that exhibit excellent sensitivity under ideal buffer conditions often show a decline in performance when operating in body fluids.^[^ ^246^, ^247^ ^]^ To maintain the sensitivity and reliability of cascade nanozyme systems during real‐sample analysis, multiple sample pretreatment strategies have been introduced, such as surface functionalization and magnetic enrichment techniques.^[^ ^151^, ^248^ ^]^ Although these approaches can enhance the specificity and anti‐interference capability of cascade nanozyme‐based sensors, they may also increase operational complexity or alter the accessibility of active sites, thereby influencing the overall sensing performance. Therefore, careful consideration should be given during the design of cascade nanozyme‐based sensing systems for practical biosensing applications. Several excellent reviews have summarized the recent progress in nanozyme‐based biosensors.^[^ ^249^, ^250^, ^251^ ^]^ Interested readers are referred to these reviews for more detailed discussions.

### 6.2 Therapy

**Figure 6:**
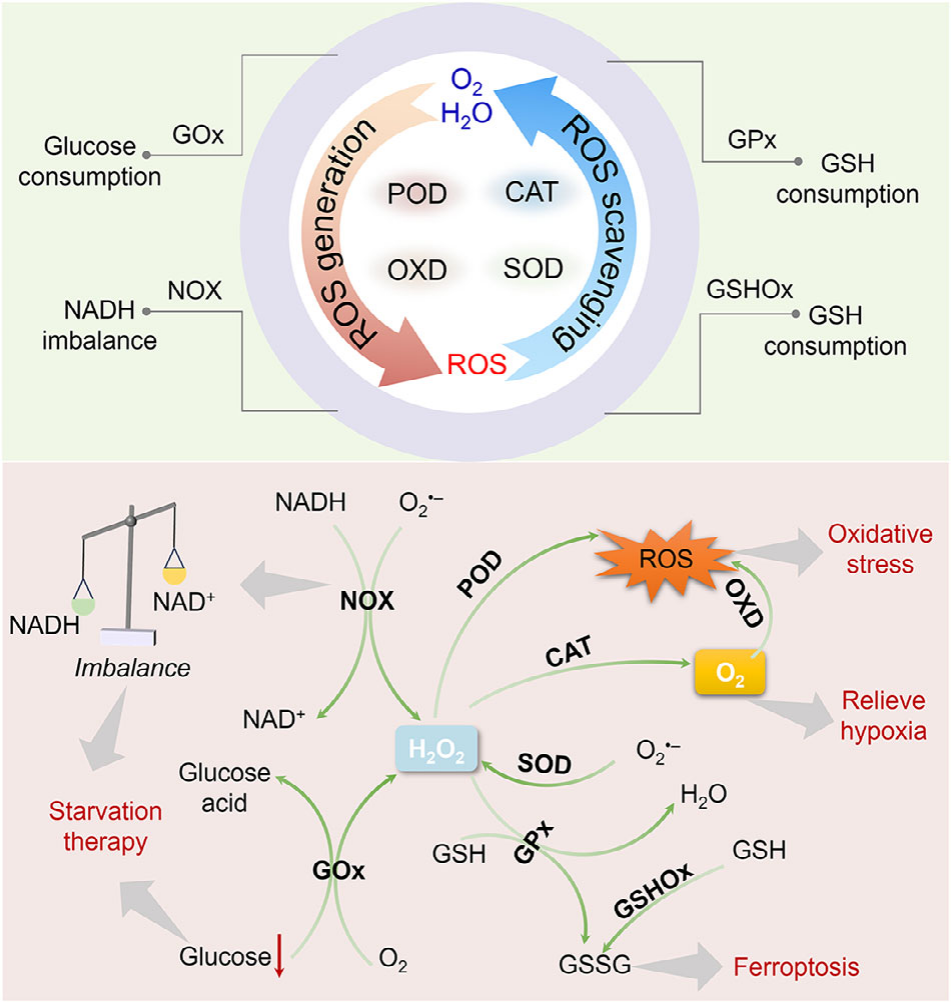
Representative model of ROS generation and scavenging via the nanozyme cascade reaction in biomedical applications.

Driven by strong catalytic performance and flexible design, all‐nanozyme cascade systems are increasingly explored for disease therapy because they can carry out multi‐step biocatalytic processes solely with engineered nanomaterials. As shown in **Figure** **6**, these systems rely on representative redox enzyme like activities, including POD, SOD, CAT, and OXD, to generate therapeutically active species or eliminate harmful molecules, thereby regulating redox homeostasis and modulating downstream biological pathways to achieve therapeutic effects. In addition, by coupling other enzyme like activities such as GSHOx, GOx, NOX, and regulating external factors such as light, radiation, and ultrasound, these systems can integrate starvation therapy, relieve hypoxia and photothermal therapy (PTT), sonodynamic therapy, and PDT, which together contribute to enhanced therapeutic efficacy. The relative robustness and tunability of nanozymes mitigate several drawbacks of natural enzymes, including short half‐life and susceptibility to proteolysis, which makes them attractive for in vivo applications. Current work focuses on the application of nanozymes in regulating the redox balance in vivo, and addressing specificity and safety challenges that still exist before clinical translation.

#### 6.2.1 Cancer Therapy

**Figure 7:**
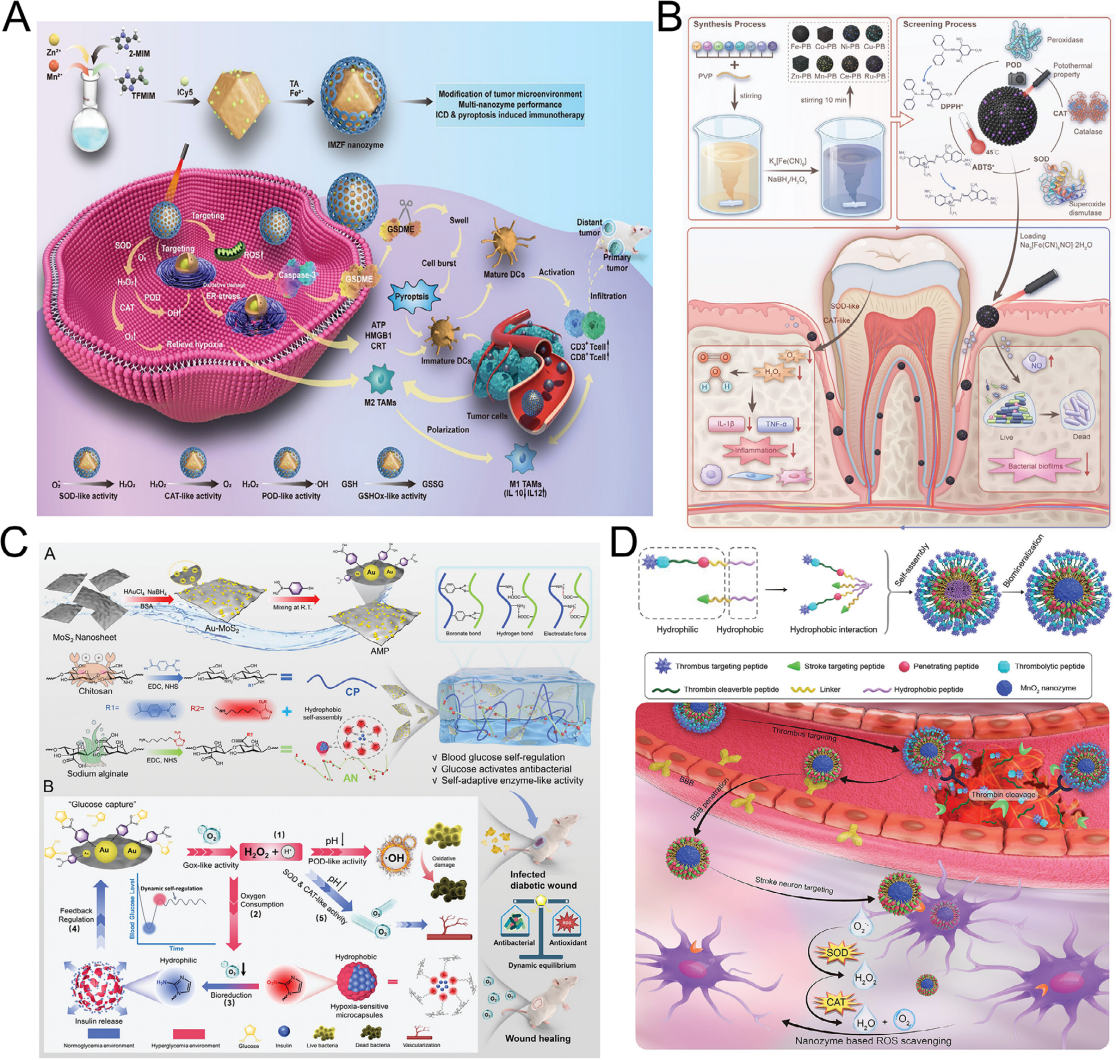
A) Preparation of IMZF and a schematic illustrating the induction of pyroptosis and ICD by IMZF for realizing immunotherapy. Reproduced with permission.^[^ ^252^ ^]^ Copyright 2024, Wiley‐VCH. B) Schematic of the development and evaluation of a nanozyme library based on Prussian blue for the treatment of periodontitis. Reproduced with permission.^[^ ^214^ ^]^ Copyright 2025, Wiley‐VCH. C) Schematic of the glucose‐activated programmed hydrogel for infected diabetic wound healing.^[^ ^232^ ^]^ Reproduced with permission. Copyright 2025, Wiley‐VCH. D) Schematic of the biomineralization synthesis of PNzyme/MnO_2_ and its application as a smart multifunctional therapeutic for the treatment of IS. Reproduced with permission.^[^ ^160^ ^]^ Copyright 2023, Wiley‐VCH.

Due to its high catalytic efficiency, multi‐enzyme cooperative interactions, and excellent adaptability to the tumor microenvironment, the nanozyme cascade system has demonstrated immense potential in cancer therapy. For example, Zhong et al. developed a multifunctional nanoscale pyroptosis inducer with cascade enzymatic activity (IMZF) (**Figure** **7**A).^[^ ^252^ ^]^ The multifunctional nanozyme can generate H_2_O_2_, O_2_, and •OH and consume glutathione, thereby altering the immunosuppressive TME. These effects not only promote M2 to M1 macrophage polarization, but also trigger immunogenic cell death (ICD)‐associated pyroptosis. Liu et al. prepared HABT‐C NPs self‐cascade nanozymes with triple‐enzyme mimetic activity.^[^ ^16^ ^]^ Its multifunctional enzymatic activities (including CAT, GOx, and POD) enable continuous O_2_ output and generation of abundant effective ROS, which can efficiently overcome hypoxia TME in tumor sites.

In addition, some nanozymes can further enhance the catalytic reaction rate, supported by their photothermal,^[^ ^172^, ^219^, ^220^, ^226^, ^231^ ^]^ ultrasonic^[^ ^24^, ^230^ ^]^ and electro‐responsive^[^ ^110^ ^]^ properties. This combination strategy demonstrates improved antitumor efficacy compared to monotherapies. For example, compared with single‐modality therapies, the integration of PTT, PDT and nanozyme‐catalytic therapy (NCT) provides a synergistic platform, which could overcome the limitations imposed by TME. Specifically, PDT relies on O_2_ to generate cytotoxic ROS. However, its therapeutic efficacy is severely limited by hypoxia conditions and elevated GSH levels of TME. In contrast, the introduction of NCT can alleviate hypoxia by the decomposition of endogenous H_2_O_2_ into O_2_ via CAT‐like activity, thereby enhancing the efficiency of PDT. At the same time, many nanozymes possess GSHOx‐like and GPx‐like activities, which deplete intracellular GSH and help maintain high ROS levels, consequently increasing tumor cell susceptibility to oxidative damage. In addition, the acidic tumors microenvironment can further promote the POD‐like activity of nanozymes and accelerate the generation of ROS. Furthermore, PTT can enhance nanozyme reaction kinetics by supplying thermal energy, thereby improving the NCT efficiency and ROS generation. As a typical example, Lin et al. developed a “tumor energy homeostasis disruptor” (Cu_2_O@Au nanozyme) for tumor treatment through the synergistic effects of starvation therapy/NCT/PTT.^[^ ^227^ ^]^ The Cu_2_O@Au nanozymes exhibited multiple enzyme‐mimicking activities (GOx, POD, and GPx), enabling it to consume glucose and GSH, and generate H_2_O_2_ and •OH for effective starvation therapy and NCT. In addition, the excellent photothermal properties of Cu_2_O@Au enable effective PTT, further enhancing NCT.

#### 6.2.2 Antibacterial Treatment

Traditional antimicrobial strategies face significant challenges, including high costs and the risk of inducing bacterial resistance. In contrast, the nanozyme cascade system offers a promising solution to these issues. By mimicking the catalytic activity of natural enzymes, nanozymes efficiently degrade bacterial biofilms and generate antimicrobial agents such as ROS, thereby enabling broad‐spectrum eradication of various pathogens. Compared to other antibacterial strategies, nanozyme‐based antibacterial methods display unique advantages, such as low cost, high stability and an approach to combat bacterial drug resistance. Since the topic of nanozyme in antibacterial therapy has been extensively reviewed in recent publications,^[^ ^253^ ^]^ this review focuses primarily on cascade nanozyme systems.

Conventional single‐activity nanozymes (such as POD‐like or OXD‐like) typically rely on ROS generation for antibacterial action. However, these systems often depend on externally supplied hydrogen peroxide as a substrate. High concentrations of H_2_O_2_ can damage healthy tissues and delay wound healing, meanwhile the hypoxic microenvironment at infection sites further limits catalytic efficiency. Cascade nanozymes overcome these drawbacks by spatially coupling two or more enzyme‐like reactions within a single nanoplatform, allowing the intermediate products of one reaction to serve as the substrates for the next. This creates a self‐sustaining catalytic cycle capable of continuous ROS production without the need for added H_2_O_2_, thereby improving both efficacy and biosafety. For example, Zhang et al. developed a multinanozyme (denoted as MnOx/q‐CeBTC) with a quasi‐MOF strategy by post‐engineering a cerium‐contained MOF (CeBTC) loaded with manganese oxide (MnOx/CeBTC).^[^ ^224^ ^]^ The resulting nanozyme exhibited excellent multiple enzymatic activities at low temperature, and enables abundant and self‐cascade ROS generation without H_2_O_2_ addition. By virtue of the ROS storm, they show superior antibacterial performance by killing *E. coil, S. aureus*, and *C. albicans* with high efficiency.

Biofilm development is widely recognized as a key contributor to persistent and drug‐refractory infections. Their dense extracellular polymeric substances (EPS) hinder antibiotic penetration and shield bacteria from host immune defenses. Cascade nanozymes with OXD‐ and hydrolase‐like activities can disrupt biofilms through a synergistic catalytic degradation mechanism, enhancing antibiotic permeability and restoring bacterial drug susceptibility. For instance, a cerium‐based MOF cascade system was shown to catalyze the cleavage of phosphoester, amide, and glycosidic bonds within biofilm matrices, followed by oxidative destruction of the biofilm structure.^[^ ^254^ ^]^ This “hydrolysis‐oxidation” cascade reaction not only promotes efficient biofilm removal but also reduces horizontal gene transfer among bacteria, thereby suppressing the spread of antibiotic resistance and achieving long‐term antimicrobial protection.

Although reactive oxygen species are crucial contributors to antimicrobial effects, their uncontrolled spread can also harm surrounding healthy tissue. Introducing stimulus‐responsive features into cascade nanozymes provides a viable strategy to improve both the precision and safety of nanozyme‐based treatments. Such systems are designed to become active only in microenvironments associated with infection—conditions like lowered pH, increased glucose concentrations, or the presence of bacterial enzymes—thereby confining their catalytic action to diseased sites and reducing collateral damage to normal tissues. For example, Xi et al. reported a nano‐scale core‐shell structure‐based oxygen‐generation system (FPB‐Co‐Ch NPs) that showed multifunctional hybrid cascaded nanozyme activities with pH‐responsive.^[^ ^255^ ^]^ After targeting the surface of *H. pylori* by electrostatic adsorption, the multifunctional cascade nanozyme activity of FPB‐Co‐Ch composite is effectively activated in the gastric acidic environment, including the oxidation of O_2_ to O_2_^•−^by OXD‐mimicking activity, then the conversion of O_2_^•−^ to H_2_O_2_ by SOD‐mimicking activity, and finally the production of large amounts of O_2_ by stronger POD‐ and CAT‐mimicking activities. In the intestinal environment, the cascade enzymatic activities are significantly inhibited, ensuring the biosafety of the treatment.

Moreover, nanozyme cascade strategy can be synergistically combined with other antimicrobial approaches, such as photothermal therapy, to further enhance its bactericidal efficacy. For instance, Fan et al. developed a ruthenium‐doped Prussian blue nanozyme (PBzyme) that exhibited multiple enzyme‐like activities, including CAT‐like, SOD‐like, and nitrogen radical scavenging activity, along with strong photothermal conversion capability (Figure 7B).^[^ ^214^ ^]^ After introducing sodium nitroprusside to create SPBzyme, this optimized and NIR‐responsive cascade nanozyme was able to effectively disrupted bacterial biofilms through mild photothermal effects, while its cascade antioxidant enzyme‐like activities suppressed oxidative stress and inflammation. The multimodal cascade platform integrates catalytic precision with spatiotemporal control, offering powerful and safe antibacterial performance.

#### 6.2.3 Wound Healing

Skin wound healing is a complex biological process that involves multiple stages, including inflammation, proliferation, and remodeling. Achieving precise regulation of the local microenvironment at each stage remains a major challenge for effective tissue repair. In this context, the application of cascade nanozyme systems offers an innovative strategy for responsive wound management. Through their multi‐enzyme cascade reactions, nanozymes can dynamically eliminate ROS and modulate oxygen supply within the wound, thereby maintaining redox homeostasis and promoting tissue regeneration. However, single nanozymes often suffer from limited catalytic stability, poor retention at the wound site, and uneven distribution, which restrict their therapeutic efficacy. The integration of nanozymes into hydrogel matrices effectively addresses these limitations. The three‐dimensional porous network of hydrogels provides a moist, conformable, and controllable release environment, while also sealing the wound, supporting sustained drug delivery, and enhancing biocompatibility. This combination significantly prolongs the catalytic lifespan and stabilizes the activity of nanozymes. Therefore, the synergistic interplay between the catalytic functions of nanozymes and the protective physicochemical properties of hydrogels has emerged as one of the most representative research directions in advanced wound healing. This section focuses on recent advances in cascade nanozyme‐hydrogel composite systems for skin wound repair. To provide a clearer overview of the design strategies and therapeutic mechanisms of cascade nanozyme‐hydrogel systems, we summarize representative examples in **Table** **3**, including their nanozyme components, cascade enzyme‐like activities, hydrogel types, main mechanisms, and therapeutic outcomes.

**Table 3:** Representative cascade nanozyme‐hydrogel systems for wound healing.

Nanozymes	Cascade steps	Hydrogel type/form	Main mechanism	Therapeutic outcomes	Refs.
SalB‐CuNCs	SOD‐CAT	Schiff‐base hydrogel (injectable)	ROS scavenging, hypoxia relief, angiogenesis	Angiogenesis; tissue regeneration	[20]
Au/Cu_1.6_O/P‐C_3_N_5_/Arg@HA (ACPCAH)	SOD‐CAT‐GOx‐POD/NOS	Ultrasound‐responsive hyaluronic acid‐based hydrogel (sprayable)	NO generation, ROS regulation, antibacterial and angiogenic effects	Wound closure; infection control	[21]
ε‐polylysine (EPL)‐coated manganese dioxide (EMn)	SOD‐CAT	NIR/glucose stimuli‐responsive CECP/OD/EMn/rGB multifunctional hydrogel (injectable)	ROS scavenging, O_2_/NO release, and glucose‐triggered Dox release	Infection control; hypoxia and oxidative stress relief; angiogenesis and collagen deposition; diabetic wound closure	[207]
Phenylboronic‐acid‐modified Au–MoS_2_ nanozyme (AMP)	GOx‐POD‐SOD‐CAT	Glucose‐responsive programmable hydrogel (adhesive)	Dynamic enzyme switching for redox regulation and insulin release	Regulation of blood sugar homeostasis; wound healing	[232]
Tannic acid‐modified ceria‐zoledronic acid nanoparticles (TCZ)	SOD‐CAT	ROS‐driven oxygenation hydrogel (sprayable)	ROS scavenging, hypoxia relief, macrophage repolarization	Diabetic wound healing; tissue remodeling	[256]
CuMnO_x_@CuO_2_@IR820 (CMCI)	POD‐CAT‐GPx	Thermosensitive low‐melting‐point agarose hydrogel (injectable)	pH‐ and temperature‐responsive ROS generation, O_2_ and H_2_O_2_ self‐supply, GSH depletion	Ablation of tumor cells and bacteria; inhibition of recurrence; angiogenesis and collagen deposition	[257]
PDA–MnO_2_ (PM)	SOD‐CAT	Bilayer hydrogel microneedle (penetrable)	ROS scavenging; photothermal‐enzyme cascade synergy	Angiogenesis; collagen deposition	[258]
CuTA NSs	SOD‐CAT	Matrix metalloproteinase‐responsive hydrogel (injectable)	ROS scavenging; macrophage repolarization	Osteogenesis and periodontal tissue regeneration	[259]

In practical research, cascade nanozyme‐hydrogel composites are typically prepared through physical embedding or chemical cross‐linking, resulting in materials that exhibit both excellent biocompatibility and physicochemical stability. These materials not only mimic the catalytic activity of natural enzymes, thereby promoting redox balance at the wound site, but also leverage the moisture‐retaining capacity of hydrogels to maintain a hydrated environment, accelerating tissue regeneration.^[^ ^257^, ^258^, ^259^, ^260^ ^]^ For example, Shang et al. designed a self‐cascade nanozyme hydrogel for tissue regeneration.^[^ ^20^ ^]^ SalB modified CuNCs (SalB‐CuNCs) not only exhibited the robust SOD‐CAT cascade catalytic performance, but also induced angiogenesis. By loading SalB‐CuNCs into a Schiff base hydrogel, the resulting cascade catalytic platform exhibited outstanding antioxidant and robust oxygenation effects in mitigating the hypoxic microenvironment.

In modern wound therapy, the role of nanozymes has evolved from a simple antibacterial agent to a multifunctional microenvironment regulator and regenerative promoter. Furthermore, stimuli‐responsive hydrogel systems enable on‐demand release of nanozymes or switching between catalytic pathways under specific local cues. For example, Guo et al. developed a glucose‐activated self‐switching enzyme‐like activity programmed hydrogel to provide self‐regulated, timely intelligent insulin release affected by blood glucose fluctuations, thereby forming a feedback blood glucose management and exerting a full‐stage wound healing (Figure 7C).^[^ ^232^ ^]^ It utilizes glucose as a sacrificial agent to generate antibacterial reactive oxygen species by recognizing the hyperglycemia environment, and releasing insulin for blood glucose regulation for up to 12 h with the help of the enzyme‐like catalysis‐generated hypoxia environment. In a normoglycemia environment, the hydrogel switches the enzyme‐like activity to supply oxygen, inhibiting further insulin release. The “environment‐sensing and function‐switching” strategies greatly enhance the stage‐specific precision control of wound repair.

The diversity of hydrogel forms has further expanded the clinical potential of cascade nanozyme systems. Sprayable hydrogels can rapidly cover large or irregular wound surfaces; injectable or self‐healing hydrogels are advantageous for filling deep wound cavities and enabling long‐term retention; and adhesive dressing hydrogels facilitate clinical application and replacement. Each form requires a balance among adhesion, release kinetics, and reversibility of crosslinking or removal. For instance, Zhu et al. developed an ultrasound‐augmented multienzyme‐like nanozyme hydrogel spray (Au/Cu_1.6_O/P–C_3_N_5_/Arg@HA, denoted as ACPCAH) by integrating ultrasound‐responsive hyaluronic acid‐encapsulated l‐arginine together with ultrasmall AuNPs and Cu_1.6_O nanoparticle‐coloaded phosphorus‐doped graphitic carbon nitride nanosheets.^[^ ^21^ ^]^ This nanozyme hydrogel spray exhibited five distinct enzyme‐mimetic activities, namely SOD‐like, CAT‐like, GOx‐like, POD‐like, and nitric oxide synthase (NOS)‐like activities. Hyaluronic acid encapsulation not only enhanced the biocompatibility and stability of nanozymes, but also decomposed specifically by hyaluronidase (HAase) in biofilms, and released l‐arginine and nanozymes to strengthen bacterial interaction. In another study, Liao et al. designed a ROS‐driven oxygenation hydrogel (OxyGel) spray that integrates a multifunctional nanozyme with a dynamically crosslinked sprayable hydrogel matrix. This nanozyme‐reinforced sprayable hydrogel, with dual ROS‐scavenging and O_2_‐generation functions, not only serves as a reactive oxygen species‐driven oxygenator to remodel the hostile inflammatory microenvironment but also protects cell proliferation, migration, and angiogenesis from oxidative damage. These findings highlight the potential of nanozyme‐based hydrogels as precision therapies adaptable to complex wound pathologies.^[^ ^256^ ^]^

#### 6.2.4 Neurodegenerative Diseases

A key factor in the pathogenesis of neurological diseases is the hyperaccumulation of ROS and reactive nitrogen species (RNS) with unpaired electrons. Elevated levels of ROS and RNS not only attack neuronal membrane lipids, DNA, and mitochondria but also induce protein misfolding and aggregation, such as α‐synuclein in Parkinson's disease and Aβ or τ proteins in Alzheimer's disease. These processes further activate glial cells and inflammatory signaling pathways, creating a vicious cycle that accelerates neuronal degeneration and death.

In this context, cascade nanozymes capable of hierarchically mimicking natural antioxidant systems, synergistically eliminating ROS and RNS, and restoring redox homeostasis are considered one of the most promising therapeutic strategies. Unlike conventional single‐activity nanozymes, cascade nanozymes integrate several enzyme‐like activities (e.g., SOD‐like, CAT‐like) within a single nanosystem. This architecture enables the sequential conversion of O_2_^•−^ into H_2_O_2_, and subsequently into water and oxygen, effectively replicating the body's intrinsic antioxidant network for continuous and hierarchical radical elimination. Therefore, cascade reactions not only enhance catalytic efficiency but also prevent the accumulation of intermediate species like H_2_O_2_ that can cause secondary toxicity. For example, Liu et al. constructed a typical nanozyme cascade antioxidant system by encapsulating dual‐Fe‐atom nanozyme (Fe_2_NC) in a selenium‐containing MOF (Se‐MOF) shell layer.^[^ ^53^ ^]^ This dual‐Fe‐atom nanozyme can be used to mimic three kinds of natural enzymes, including SOD, CAT, and GPx. Therefore, it can protect middle cerebral artery occlusion rats with decreased infarct volume and improved neurological deficits by scavenging excessive ROS, ameliorating oxidative‐stress induced injury, and suppressing cell apoptosis.

Moreover, some recent works have elucidated the underlying cellular and synaptic mechanisms by which cascade nanozymes confer neuroprotection. For example, Mao et al. reported a Fe single‐atom nanozymes (Fe_1_/NC SAzymes), possessing both CAT‐like and SOD‐like activities, could effectively alleviate Parkinson's disease symptoms by rebalancing neuronal redox homeostasis.^[^ ^261^ ^]^ In a 1‐methyl‐4‐phenylpyridinium (MPP^+^)‐induced PD model, the nanozyme not only scavenged excess ROS but also restored neurotransmission functions. In vivo experiments showed that Fe_1_/NC SAzyme improved dopamine release, rescued dopaminergic neurons, and reduced motor deficits. At the single‐cell level, single‐vesicle electrochemical analyses further revealed that the nanozyme mitigated MPP^+^‐induced damage by enhancing vesicular neurotransmitter release and regulating fusion pore dynamics. This study provides compelling evidence for the application of cascade nanozyme systems as neuroprotective agents and highlights the importance of redox regulation at both cellular and synaptic levels in neurodegenerative disease treatment.

In fact, the therapeutic effect of cascade nanozymes in the brain depends on their ability to cross the blood‐brain barrier (BBB). The challenge has led to surface modification and carrier optimization of cascade nanozyme, becoming the key research direction in the field of neurodegenerative diseases. Recent reports demonstrate that conjugating nanozymes with brain‐targeting ligands such as transferrin‐receptor ligands (or T7 peptide) and lactoferrin can promote active transcytosis and targeted brain delivery.^[^ ^262^, ^263^, ^264^ ^]^ For example, a peptide‐templated manganese dioxide nanozyme (PNzyme/MnO_2_) was rationally designed to cross the BBB and exert both thrombolytic and neuroprotective effects in ischemic stroke (IS) (Figure 7D).^[^ ^160^ ^]^ This nanozyme was templated by self‐assembled polypeptides incorporating multiple functional motifs, including a T7 peptide (HAIYPRH) that bound to transferrin receptors on endothelial cells to mediate BBB penetration, and a stroke‐homing peptide (CLEVSRKNC) targeting apoptotic neurons. Upon reaching the ischemic brain regions, the nanozyme bound to fibrin via a CREKA motif and was enzymatically cleaved by thrombin to release the thrombolytic peptide GRPAK, thereby initiating localized thrombolysis. Simultaneously, its MnO_2_ component mimicked SOD and CAT activities, efficiently scavenging reperfusion‐induced ROS to protect neuronal tissues and reduce inflammation.

Overall, cascade nanozymes have been shown to synergistically regulate oxidative stress, inflammation, and protein aggregation through multiple mechanisms, making them an important frontier in the research of neurodegenerative disease treatment. With the further improvement of material design, delivery technology, and biological safety assessment, the emerging cascade system is expected to provide more efficient and safe treatment strategies for neurodegenerative diseases.

#### 6.2.5 Challenges and Opportunities of Cascade Nanozymes Systems in Therapy

Cascade nanozyme systems face multiple biocompatibility challenges during therapeutic application, including biological barriers, immunogenicity, protein corona formation, and evolving disease microenvironments. These processes may substantially alter nanoparticle surface properties, biodistribution, and catalytic performance. To reduce these effects, various surface engineering strategies have been developed to construct cascade nanozyme systems, such as liposomal encapsulation and natural cell membrane coating.^[^ ^47^, ^265^ ^]^ These approaches not only facilitate immune evasion and traversal of biological barriers but also provide antifouling interfaces that limit protein corona formation, thereby preserving catalytic site accessibility and maintaining enzymatic activity. Moreover, tailoring coating architecture and ligand decoration can further enhance targeting capability and promote accumulation within tumor microenvironments,^[^ ^91^ ^]^ thereby improving the bioavailability and therapeutic selectivity of cascade nanozymes. In addition, in the evolving disease microenvironments, some cascade nanozymes can dynamically adjust their cascade reaction pathway according to local biochemical cues, such as acidity, oxidative stress, or metabolic state.^[^ ^16^, ^139^, ^232^ ^]^ More importantly, after being rationally designed, these microenvironment‐sensitive cascade nanozymes could exploit the dynamic characteristics of tumors to achieve enhanced therapeutic outcomes.

On the other hand, the biological safety of cascade nanozymes remains the main obstacle for clinical translation. For example, during in vivo diagnostic or therapeutic applications, the evolving disease microenvironment may induce nanozyme inactivation, metal ion dissolution, or off‐target toxicity. This phenomenon threatens the efficacy and safety of nanozymes in the human body and significantly limits their clinical application and scalability. Moreover, given the intrinsic propensity of nanozymes to accumulate in organs such as the liver, lungs, and spleen, in vivo dosing should be optimized to balance catalytic efficacy with dose‐dependent toxicity. The potential accumulation and persistence of non‐degradable nanoparticles may trigger chronic toxicity and immune responses. To address these concerns, researchers have made progress in improving the biosafety of nanozymes by various rational design strategies. These include synthesizing nanozymes with ultra‐small sizes that can be cleared by the kidneys to enhance their clearance rate, using gold‐based nanozymes with high biocompatibility and stability; and developing biodegradable nanomaterials (such as organic polymers) to promote degradation and metabolic clearance. Systematic discussions on the safety of therapeutic nanozymes have been extensively covered in a relevant review.^[^ ^266^ ^]^ Interested readers can refer to the review to obtain a more comprehensive and detailed discussion.

In summary, the biosafety and biocompatibility of cascade nanozyme systems are critical obstacles to clinical use. Although some cascade nanozymes have been reported with good biocompatibility, their application in vivo requires further optimization. In addition, current toxicity assessments are often limited to cell experiments or small animal models, but these are far removed from human clinical applications. There is a current lack of mechanistic understanding of interactions with normal issues and the immune systems. The risks posed by slow degradation, metal‐ion leaching, and chronic inflammation underscore the need for comprehensive long‐term toxicology and pharmacokinetic studies. Only by addressing these multifaceted challenges can cascade nanozyme systems achieve safe, effective, and controllable in vivo performance suitable for therapeutic deployment.

## 7 Conclusion and Prospects

Owing to their remarkable catalytic efficiency and inherent signal amplification capabilities, enzyme cascade systems have attracted considerable attention in various biomedical fields, particularly in disease therapy and biosensing. However, the practical application of natural enzymes remains substantially limited by their stability, bioavailability, and cost limitations. The emergence and evolution of nanozymes have provided powerful alternatives and novel design paradigms for constructing whole nanozyme‐based cascade systems. Benefiting from unique structural stability, designability, and multi‐enzyme activity of nanozyme, these nanozyme‐constructed cascades, offer notable improvements in catalytic activity, substrate selectivity, operational stability, and signal transduction efficiency.

In recent years, the development of engineered all‐nanozyme cascade platforms has progressed rapidly, with widespread applications in diverse areas, including biosensing, cancer therapy, antibacterial treatments, wound healing, and neurological disorder management. This review systematically summarized the structural design principles underlying cascade catalysis, emphasized the evolution from self‐cascading nanozyme systems to multi‐component immobilized architectures, and categorized representative nanozyme cascade models. Additionally, this review highlighted recent advances in their biomedical and bioanalytical applications. Nevertheless, several challenges remain to be addressed to accelerate the rational design and practical translation of nanozyme‐based cascade systems:

1) Deeper exploration of cascade reaction in confined environments. The advantages of enzyme cascade reactions are typically manifested in high local concentration, efficient mass transfer, and reduced intermediate product decomposition, all of which contribute to enhanced catalytic performance. However, these benefits are often not fully realized in open environments. For example, in some cases, the catalyst used in the previous step must be removed, leading to the loss of materials and consequently affecting the overall reaction efficiency. In contrast, cascade reactions in confined environments better simulate the catalytic mechanisms of biological systems, where the local substrate concentration and reaction effectiveness can be more easily controlled, preventing material loss and incomplete conversion of intermediate products. Therefore, future research should focus on constructing cascade reaction systems in restricted environments to achieve higher reaction efficiency and stability.
2) Self‐cascading reaction through the exploitation of multienzyme activities at the same active site, reducing material loss and making material transport simple and efficient. This design avoids the need to synthesize multiple enzymes, simplifying the synthesis process and improving experimental operability and cost‐effectiveness. Although multi‐enzyme activity of self‐cascading nanozyme can construct the cascade reaction, the catalytic efficiency is often affected by competitive or antagonistic effects. It means that the overall catalytic activity is not merely the sum of individual enzyme activities, but rather a complex interaction of various factors. Therefore, to achieve an efficient and functionally controllable multi‐enzyme nanozyme catalyst system, it is essential to deepen the theoretical understanding of its mechanism and develop new strategies to accelerate the efficient and directional synthesis of multi‐enzyme nanozymes.
3) Compared with self‐cascading nanozymes, the immobilized nanozyme cascade system can integrate different activities of nanozymes according to needs, which maybe become the development trend of constructing nanozyme cascade system. However, during the process of nanozyme immobilization, the active site may be covered or lost, thus affecting the activity of the immobilized enzyme.^[^ ^267^ ^]^ Therefore, when constructing these cascade nanozymes, it is necessary to balance the relationship between the immobilization strategy and activity of enzyme‐like activity to achieve the best catalytic efficiency.
4) Although various cascade nanoreactors based on nanozymes have been developed, their overall catalytic performance in complex systems has often fallen short of expectations. The main challenge lies in the fact that different nanozymes exhibit optimal activity under distinct conditions of solvent composition, pH, and temperature. When multiple nanozymes are assembled within a single carrier or reactor, compromises between these requirements are unavoidable, preventing each step from operating under its ideal conditions. In living systems, cells overcome this limitation by compartmentalizing enzymes within membrane‐bound organelles or phase‐separated structures. Compartmentalization prevents interference between incompatible reactions, such as aerobic and anaerobic steps or processes that require very different pH or redox conditions. It also allows each reaction to be optimized within its own microenvironment. These natural strategies provide important inspiration for improving the efficiency of nanozyme cascades. To enhance cascade performance, future research may focus on the controlled synthesis of nanoreactors with hierarchical pore structures and regionally differentiated interfacial properties, allowing the precise spatial organization of nanozymes with distinct catalytic activities. By confining each nanozyme within its own optimized microenvironment, it may be possible to achieve significantly higher overall cascade efficiency.
5) Compared with natural enzymes, nanozymes generally suffer from limited catalytic specificity, which is a major bottleneck in cascade nanozyme systems. The intrinsic low substrate selectivity of nanozyme often causes undesired side reactions, especially in the cascade nanozyme system. This limitation arises mainly from the absence of well‐defined active pockets, resulting in poor substrate recognition. Although biomimetic substrate‐channeling strategies have been developed to improve selectivity by stepwise filtering of intermediates,^[^ ^22^ ^]^ these designs are usually system‐specific and difficult to generalize. Enhancing selectivity is also essential for ensuring in vivo safety, as low biological specificity may lead to off‐target toxicity and reduced bioavailability, ultimately necessitating higher therapeutic doses.

In addition to selectivity, kinetic mismatch among cascade steps is another major challenge. For instance, typical GOx‐POD cascade systems suffer from pH and rate incompatibility – the slow accumulation of gluconic acid would delay the reaction of POD‐like nanozymes. Furthermore, the catalytic activity and stability of nanozymes under complex physiological conditions are still poorly understood. Most current studies rely on simplified buffer systems, which cannot accurately reflect the heterogeneous microenvironments, fluctuating pH, ionic strength, and competing biomolecules encountered in vivo. These factors can significantly alter nanozyme activity and selectivity, leading to discrepancies between in vitro and in vivo performance. Future efforts should focus on catalytic behaviors of nanozyme under physiologically relevant conditions, and systematically evaluate their catalytic efficiency, stability, and substrate specificity within complex biological matrices containing proteins, lipids, and nucleic acids.

6) Cascade nanozymes are currently at a critical stage of transition from fundamental research to clinical application. Their future development will rely on the integration of artificial intelligence technologies and systematic biological assessment. The use of machine learning and high‐throughput computational methods enables precise optimization of active‐site configurations, simulation of catalytic pathways, and prediction of structure–activity relationships. The interdisciplinary collaborative approach can improve both efficiency and functional controllability by establishing a data‐driven, intelligent design framework. Furthermore, before clinical translation, strict in vivo safety assessment is required, including detailed investigations of biodistribution, metabolic fate, immunogenicity, and long‐term toxicity. Therefore, future studies should focus on developing standardized assessment models and integrating artificial intelligence‐based predictive analyses. This will promote application of nanozyme in dynamics and complex physiological environments and support their safe, effective use in precision medicine.

## Conflict of Interest

The authors declare no conflict of interest.
